# Transcriptomic profiling of Indian breast cancer patients revealed subtype-specific mRNA and lncRNA signatures

**DOI:** 10.3389/fgene.2022.932060

**Published:** 2022-10-25

**Authors:** Meghana Manjunath, Snehal Nirgude, Anisha Mhatre, Sai G. Vemuri, Mallika Nataraj, Jayanti Thumsi, Bibha Choudhary

**Affiliations:** ^1^ Institute of Bioinformatics and Applied Biotechnology, Bengaluru, India; ^2^ Manipal Academy of Higher Education, Manipal, India; ^3^ Division of Human Genetics,Children’s Hospital of Philadelphia, Philadelphia, PA, United States; ^4^ BGS Global Hospital, Uttarahalli Main, Bengaluru, India

**Keywords:** transcriptomics, long noncoding (lnc) RNA, breast cancer, gene expression, overall survival

## Abstract

Breast cancer (BC) is one of the leading causes of cancer-associated death in women. Despite the progress in therapeutic regimen, resistance and recurrence of breast cancer have affected the overall survival of patients. The present signatures, such as PAM50 and Oncotype DX, do not segregate the Indian breast samples based on molecular subtypes. This study aims at finding signatures of long noncoding RNA (lncRNA) and mRNA in Indian breast cancer patients using RNA-seq. We have analyzed the survival based on the menopausal and hormone status of 380 Indian breast cancer patients, and of these, we have sequenced and analyzed matched tumor–normal transcriptome of 17 (pre- and postmenopausal) Indian breast cancer patients representing six different subtypes, namely, four patients in triple-positive, three patients in estrogen receptor–positive (ER+ve), three patients in estrogen and progesterone receptors–positive (ER+ve, PR+ve), two patients in human epidermal growth factor receptor (Her2+ve), three patients in triple-negative, and one patient in ER+ve and Her2+ve subtypes. We have identified a 25 mRNA–27 lncRNA gene set, which segregated the subtypes in our data. A pathway analysis of the differentially expressed genes revealed downregulated ECM interaction and upregulated immune regulation, cell cycle, DNA damage response and repair, and telomere elongation in premenopausal women. Postmenopausal women showed downregulated metabolism, innate immune system, upregulated translation, sumoylation, and AKT2 activation. A Kaplan–Meier survival analysis revealed that menopausal status, grade of the tumor, and hormonal status displayed statistically significant effects (*p* < 0.05) on the risk of mortality due to breast cancer. Her2+ve patients showed low overall survival. One of the unique lncRNA-mRNA pairs specific to the EP-subtype, SNHG12 and EPB41, showed interaction, which correlates with their expression level; SNHG12 is downregulated and EPB41 is upregulated in EP samples.

## Introduction

Breast cancer accounts for 25% of all cancers and exhibits heterogeneity with varied molecular and clinical characteristics (Hwang et al., 2019). The incidence and mortality rates for breast cancer, according to GLOBOCAN in 2020, were 34,65,951 new cases and 11,21,413 deaths worldwide and 1,204,532 new cases and 436,417 deaths in India (Sung et al., 2021), respectively.

Breast cancer is broadly classified based on a hormonal status analysis using immunohistochemistry as luminal A [progesterone receptor (PR)–positive, estrogen receptor (ER)–positive, and human epidermal growth factor 2 (Her2)–negative] and luminal B (ER-positive, PR-positive/negative, and Her2-positive) being the estrogen-positive subtypes, Her2 enriched, and triple-negative breast cancer (Perue et al., 2000; Yeh and Mies, 2008; Penault-Llorca and Viale, 2012). The fifth subtype is normal-like, resembling normal breast tissue features. Another distinctive subtype that shows lower claudin, epithelial to mesenchymal markers, and immune receptor expression has been recently identified using molecular analysis (Fedele et al., 2017; Manjunath and Choudhary, 2021).

Gene expression signatures have been used in the past decade for prognosis and to guide treatment in hormone-positive breast cancer patients (Villarreal-Garza et al., 2020). Oncotype DX, MammaPrint, and prediction analysis of microarray 50 (PAM50) are some commercially available genomic signatures used in the clinics (Paluch-Shimon et al., 2017; Andre et al., 2019; Cardoso et al., 2019). MammaPrint categorizes patients by low and high risks based on the 70-gene profile from the microarray (Van De Vijver et al., 2002; Van’t Veer et al., 2002). Oncotype DX is based on the 21 gene expression from the FFPE samples. The relative expression of these genes gives a recurrence score, grouping patients into low, intermediate, and high risk (Paik et al., 2004; Sparano et al., 2015). Prosigna or the PAM50 test depends on the expression of a 50-gene panel that distinguishes the tumor into molecular subtypes and provides the risk of recurrence score (ROR) (Parker et al., 2009; Nielsen et al., 2014). However, these tests have shown success only in Caucasian postmenopausal patients and not in younger women with the disease (Paluch-Shimon et al., 2017; Andre et al., 2019). Also, these sets have been shown to segregate samples only in the microarray data and not in the RNA-seq data.

Our understanding of the molecular features of cancer has been revolutionized due to recent advances in next-generation sequencing technology (Casamassimi et al., 2017), enabling global profiling of mRNAs and noncoding RNAs such as long ncRNAs (lncRNAs), microRNAs, and circular RNA. lncRNAs have now been well studied in gene regulation and are known to participate in the development and prognosis of cancer (Prensner and Chinnaiyan, 2011; Huarte, 2015; Rao et al., 2017). Specific mRNA and lncRNA signatures have been associated with different molecular subtypes of breast cancer (Deva Magendhra Rao et al., 2019). An Indian cohort study on 543 patients showed that 47% of the BC patients were below 50 years of age. In addition, 60% of the cohort presented HER2+ or TNBC disease (Thumsi et al., 2014). The advanced stages of the disease, 51% and 45% of stage III and stage IV, belonged to the HER2+ subtype. Recurrence was most frequently observed in HER2+ and TNBC (Thumsi et al., 2014). In the present study, a survival analysis coupled with Cox has been performed to find the prognostic markers. The Kaplan–Meier log-rank test and Cox proportional hazard regression are powerful and widely used survival analyses approach (Therneau and Grambsch, 2000; Karrison, 2016). The molecular heterogeneity of the Indian cohort has not been explored in the subtypes of breast cancer. This study aims to identify signatures that can stratify BC patients and guide their therapy based on altered pathways; furthermore identifying lncRNA-mRNA regulatory pairs and analyzing the probable mechanism of lncRNA involvement in breast cancer progression using *in silico* tools.

## Methodology

### Study cohort and sample classification

The breast cancer patient samples used for the study were procured from the BGS Global Hospital, Bengaluru, Karnataka, India. The tumor tissue (*n* = 17) and their respective matched normal (*n* = 16) samples were later collected in RNA, accounting for a total of 33 samples. TRIzol was added to the samples and stored at −80 until further processing. The samples obtained for the study were histologically classified as invasive ductal carcinoma (IDC) (except for one sample, which was mucinous). The obtained 17 breast cancer patient samples could be classified into six different subtypes based on the expression of estrogen, progesterone, and Her2, which are summarized in Table 1. Samples and matched normal samples were also used as a validation cohort. The study was performed under ethical approval from the BGS Global Hospitals and IBAB (IEC/Approval/2018-05/06/01A).

**TABLE 1:** Table depicting sample details of Indian breast cancer patients. Odd numbers are matched normals and even numbers are tumor samples. There are a total six subtypes (ER, EH, EP, EPH, Hmod, and TNBC) classified based on the expression of estrogen receptor (ER), progesterone receptor (PR), and epidermal growth factor receptor (Her2). IDC, invasive ductal carcinoma.

Patient No.	Sample	Label	Age (years)	Menopause status	ER	PR	Her2	Subtype label	Type	Recurrence	Status	Ki67	Grade
Patient 1	Normal	P1	56	Pre	Positive	Positive	Positive	EPH	IDC	No	Alive	Low	II
	Tumor	P2											
Patient 2	Normal	P3	56	Pre	Positive	Positive	Positive	EPH	Mucinous	No	Alive	Low	II
	Tumor	P4											
Patient 3	Normal	P5	41	Pre	Negative	Negative	Negative	TNBC	IDC	No	Alive	High	II
	Tumor	P6											
Patient 4	Normal	P7	41	Pre	Positive	Negative	Negative	EP	IDC	No	Alive	Low	II
	Tumor	P8											
Patient 5	Normal	P9	62	Post	Positive	Negative	Negative	ER	IDC	No	Alive	Low	II
	Tumor	P10											
Patient 6	Normal	P11	38	Pre	Positive	Negative	Negative	ER	IDC	No	Alive	NA	NA
	Tumor	P12											
Patient 7	Normal	P13	48	Pre	Positive	Negative	Negative	EH	IDC	No	Alive	High	II
	Tumor	P14											
Patient 8	Normal	P15	48	Pre	Positive	Negative	Negative	EH	IDC	Yes	Alive	Low	NA
	Tumor	P16											
Patient 9	Normal	P17	29	Pre	Negative	Negative	Negative	TNBC	IDC	No	Alive	High	II
	Tumor	P18											
Patient 10	Normal	P19	50	Post	Negative	Negative	Positive	Hmod	IDC	No	Alive	Low	NA
	Tumor	P20											
Patient 11	Normal	P21	35	Pre	Positive	Positive	Positive	EPH	IDC	Yes	Alive	Low	II
	Tumor	P22											
Patient 12	Normal	P25	60	Post	Negative	Negative	Positive	Hmod	IDC	No	Alive	High	II
	Tumor	P26											
Patient 13	Normal	P27	60	Post	Negative	Negative	Negative	TNBC	IDC	No	Alive	High	II
	Tumor	P28											
Patient 14	Normal	P29	58	Post	Positive	Negative	Negative	ER	IDC	No	Alive	High	II
	Tumor	P30											
Patient 15	Normal	P42	60	Post	Positive	Positive	Negative	EP	IDC	No	Alive	NA	II
Patient 16	Normal	P43N	65	Post	Positive	Positive	Negative	EP	IDC	No	Alive	NA	II
	Tumor	P43T											
Patient 17	Normal	P44N	72	Post	Positive	Positive	Negative	EPH	IDC	No	Alive	NA	II
	Tumor	P44T											

### RNA isolation and library preparation

Total RNA was extracted using the standard TRIzol method from matched tumor and normal samples. RNA was quantitated using QUBIT, and the quality was checked using TapeStation. mRNA libraries were prepared using Illumina TruSeq RNA Library Prep Kit v2.

In brief, mRNA was isolated using oligo-dT beads, followed by fragmentation. Fragmented RNA was then converted to cDNA, and adaptor ligation was performed. Size selection was performed on adaptor-ligated libraries using AMPure beads. The libraries were amplified and checked on a tape station to determine the library size.

### RNA sequencing and data analysis

The samples were sequenced in-house using Illumina HiSeq 2500 to acquire 100 bp paired-end reads. Samples had reads >10 million (Supplementary Table S1). The quality of the reads was checked using the FastQC tool (Wingett and Andrews, 2018). The reads were quantile normalized using the normalize.quantiles function in R. The reads were then aligned to the reference hg38 [downloaded from the University of California, Santa Cruz (UCSC) genome browser] using bowtie2 with default parameters (Langmead and Salzberg, 2012). A sequence alignment map (SAM) format file was obtained as an output of bowtie2. A binary alignment map (BAM) file was obtained using SAMtools (Li et al., 2009) from the SAM file. The hg38refseq.bed annotation file was downloaded from UCSC, and read counts were generated using bedtools (Quinlan and Hall, 2010). The read counts for each matched normal and tumor pair were given as the input to DESeq, an R package to obtain differentially expressed genes (DEGs) (Anders and Huber, 2010). Groupwise differential gene expression was performed for each subtype between normal and tumor samples using DESeq2.

### Pathway enrichment analysis

A cutoff of *p*-value less than 0.05 and log2 fold change (<−1 and >+1) was used to obtain a significant DEG list for each normal tumor sample pair. For groupwise DESeq2 among the subtypes, FDR-corrected *p*-value less than 0.05 and log2 fold change (<−1 and >+1) were put as cutoff. Significant DEGs common to all patients in a subtype were taken out and subjected to the Reactome pathway analysis (https://reactome.org/) to obtain subtype-specific upregulated and downregulated signature pathways. Also, premenopause and postmenopause signature pathways, and pathways with a false discovery rate of less than 0.1 have been plotted in a bubble plot using ggplot2, an R package.

### lncRNA analysis

The Bam files obtained for each tumor and their respective matched normal samples from SAMtools were given as an input to bedtools with the gencode.v34.long_noncoding_RNAs.gtf annotation file obtained from GENCODE (https://www.gencodegenes.org/human/release_34.html). The read count file for each tumor–normal pair was given as the input to DESeq (R package) to obtain differentially expressed (DE) lncRNAs. lncRNAs were then compared against the Lnc2cancer database (Ning et al., 2016), and the known breast cancer–related lncRNAs were selected.

The bedtools intersect function was used to screen for overlaps between two sets of genomic features. To obtain lncRNA-mRNA pairs for each subtype, the list of unique lncRNAs with information on the genomic regions and the NCBI RefSeq hg38 reference was given as the input to bedtools intersect. To generate potential overlapping (antisense) lncRNA-mRNA pairs, a window of greater than 1,000 bases was selected.

### Euclidean distance calculation

The “dist” function in R was used to calculate the Euclidean distance between samples. A principal component analysis (PCA) was performed on all patient samples using the PCA function of the DESeq2 plot with the different subtypes as the variables of interest. Significant genes from different patient samples with DEGs were sorted based on the *p*-value and log2 fold change. A heat map was plotted for the filtered genes using the pheatmap function with default Euclidean distance parameters. Hierarchical clustering was performed to determine the overall similarity and signature of breast cancer patient subtypes using gene expression profiles and was visualized using the pheatmap function.

### Survival analysis

To investigate the impact of the clinical parameters, such as menopausal status, age, stage, and grade of tumor, and therapy on the prognostic survival of breast cancer patients, a KM survival curve analysis was carried out and hazard ratio (HR) and 95% confidence intervals (CIs) were estimated by using the Cox proportional hazards regression model. Clinical parameters of 381 Indian breast cancer patients were obtained from the BGS Global Hospital (out of which 17 samples were sequenced). Univariate and multivariate Cox analyses were carried out with survminer (https://github.com/kassambara/survminer) and survival packages of R (Therneau and Grambsch, 2000).

### Extraction of breast cancer expression data from The Cancer Genome Atlas

The RSEM values for 946 breast cancer patient files (BRCA.rnaseqv2 illuminahiseq_rnaseqv2 unc_edu Level_3 RSEM_genes_normalized data.data.txt) were downloaded from The Cancer Genome Atlas (TCGA; http://firebrowse.org/?cohort=BRCA#). The file having barcode information for each patient was also procured from TCGA (https://portal.gdc.cancer.gov/) to obtain hormone receptor subtype information.

### LASSO regression model

The LASSO regression for the 25 genes obtained from our Indian cohort data was performed in TCGA samples, as described previously (Desai et al., 2021). In brief, LASSO-Cox regression was used with the data set to predict the possible features responsible for a death event using sksurv and scikit-survival modules of scikit-learn in Python. We performed this regression to obtain a model, with significant genes and their coefficient values with respect to the death event. These coefficient values were used with the respective gene values to estimate the risk score and perform a survival analysis using the Kaplan–Meier estimate in R library packages survival and survminer. The immune profiling was performed for the gene signature obtained from the LASSO model using CIBERSORT (Chen et al., 2018). Also, drug–gene interactions were predicted using DGIdb (The Drug Gene Interaction Database) (Cotto et al., 2018) for the LASSO model gene signatures.

### First-strand cDNA synthesis

Once the intact RNA was obtained, complementary DNA (cDNA) synthesis was initiated. For synthesizing cDNA from mRNA, random hexamers were used. A total of 4 µg of RNA was taken from each patient sample from the validation cohort for making cDNA. To remove DNA contamination, the RNA samples were treated with DNase I (37°C, 10 min) and cDNA was synthesized using M-MuLV reverse transcriptase (37°C, 1 h). Initially, the RNA samples were incubated with adaptor primers and dNTPs for 1 h at 37°C (Tzanetakis et al., 2005). A reaction without reverse transcriptase (RTase) was kept as a negative control for each sample.

### Real-time polymerase chain reaction for investigating the expression of marker genes

Real-time PCR was conducted using SYBR^®^ Green chemistry (Ponchel et al., 2003). BCL2, BRCA1, TP53, CD44l, CD44s, ALDH1A, and HOTAIR genes were used with GAPDH primer as an internal control. The sequences of the primers are described in the Table 2. The initial denaturation was at 95°C for 5 min, followed by 40 cycles of 95°C for 20 s, 53°C–55°C for 20 s, and 72°C for 20 s, and a melt curve analysis was carried out. Here, the relative gene expression was calculated by correlating the expression of the housekeeping gene and the expression of the target gene in the control/normal sample (Deepak et al., 2007). Ct is the cycle number at which the fluorescence crosses the threshold level (Livak and Schmittgen, 2001; Schmittgen and Livak, 2008). The equation for relative quantitation (RQ) value is

$${RQ=2}^{-\Delta \Delta Ct}$$

where

$$∆∆Ct=∆{Ct\;}_{\left(Tumor\;sample\right)}-∆{Ct\;}_{\left(Normal\;sample\right)},$$

and

$$\Delta {Ct\;}_{\left(Normal\;sample\right)\;}{=Ct\;}_{\left(target\;gene\;of\;normal\;sample\right)\;}{-Ct\;}_{\left(housekeeping\;gene\;of\;normal\;sample\right),}$$

$$\Delta {Ct\;}_{\left(Tumor\;sample\right)}{=Ct\;}_{\left(target\;gene\;of\;tumor\;sample\right)}{-Ct\;}_{\left(housekeeping\;gene\;of\;tumor\;sample\right)}$$

**TABLE 2:** Sequences of primers used for RT-PCR validation.

Gene	Primer sequence	
	Forward	Reverse
GAPDH	5’-CCC​TTC​ATT​GAC​CTC​AAC​TAC​AT-3’	5’-CTG​GAG​ATG​GTG​ATG​GGA​TTT-3’
BCL2	5’-AGA​GAC​TCA​CCA​GGG​TCT​GC-3’	5’-GCA​CTA​CCT​GCG​TTC​TCC​TC-3’
BRCA1	5’ CTG​CCG​TCC​AAA​TTC​AAG​AAG​T-3’	5’-CTT​GTG​CTT​CCC​TGT​AGG​CT-3’
TP53	5’-CTG​CTT​GCC​ACA​GGT​CTC-3’	5’-TGG​ATG​GGT​AGT​AGT​ATG​GAA​G-3’
ALDH1A	5’-ACT​TAC​CTG​TCC​TAC​TCA-3’	5’-GGATGAAGGTCCTGCTTTCCTT-3’
CD44l	5’-CAG​GTG​GAA​GAA​GAG​ACC​CAA​A-3’	5’-GGA​TGA​AGG​TCC​TGC​TTT​CCT​T
CD44s	5’-TCC​AAC​ACC​TCC​CAG​TAT​GAC​A-3’	5’-GGC​AGG​TCT​GTG​ACT​GAT​GTA​CA-3’
HOTAIR	5’-GGT​AGA​AAA​AGC​AAC​CAC​GAA​GC-3’	5’-ACA​TAA​CCT​CTG​TCT​GTG​AGT​GCC-3’

Graphs showing relative quantification for all the samples were plotted using the GraphPad Prism software (Swift, 1997).

### Statistical analysis

Statistical analyses and graphing were carried out using GraphPad Prism 7.0 software (GraphPad, San Diego, CA, United States) and R packages. DESeq2 uses the Wald test statistic with a probability to generate a significant gene list. The Benjamini–Hochberg false discovery rate (FDR) method was used for choosing significant pathways from the Reactome database. For a comparative qRT-PCR analysis, a two-tailed *t*-test was applied to calculate the significance. If the *p*-value was less than 0.05, the results were significant.

## Results

### Her2-positive patients and recurrent disease subgroup had poor survival among breast cancer subtypes in Indian cohort

**FIGURE 1:**
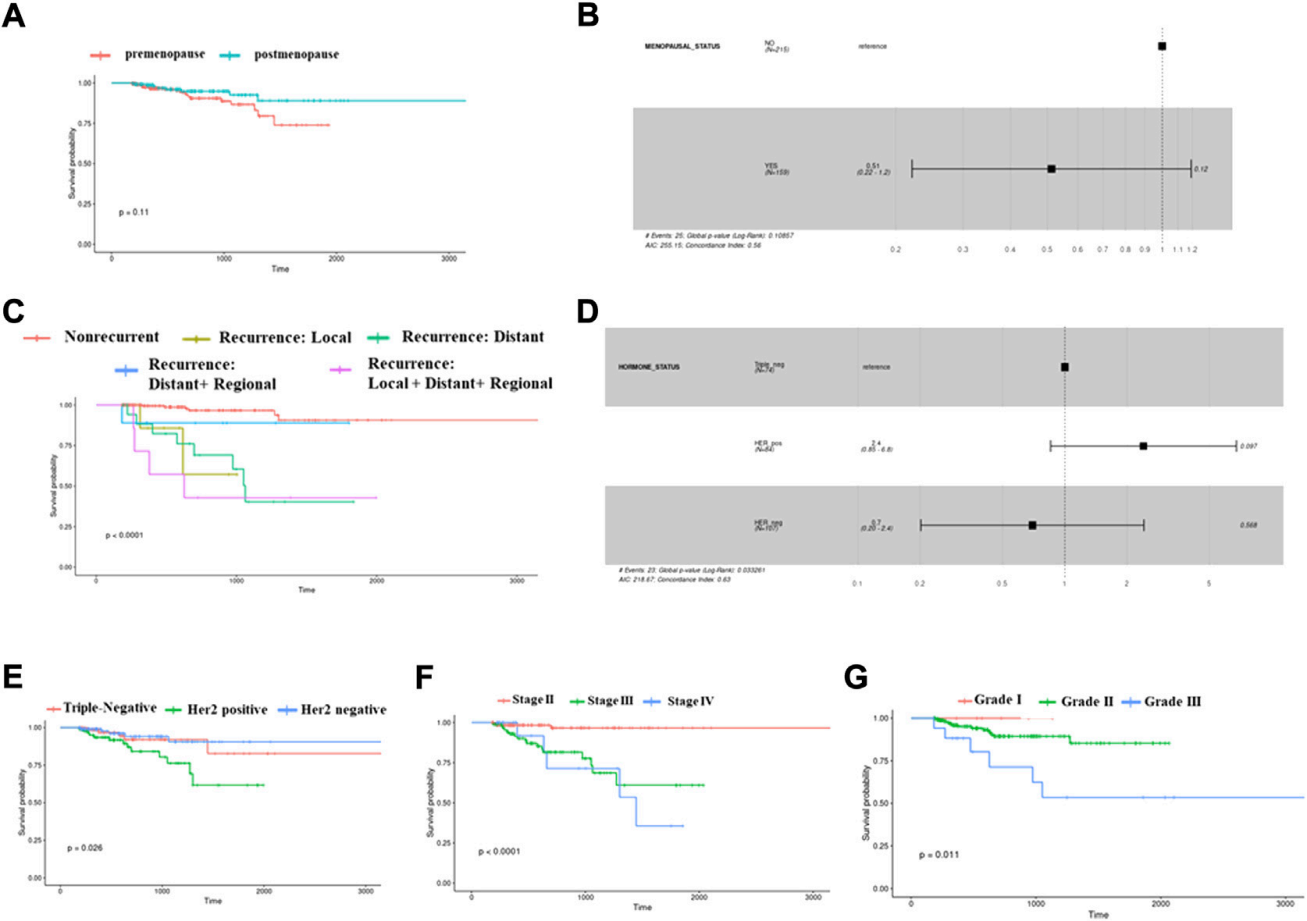
Kaplan–Meier survival plots showing differences in probabilities between various clinical parameters. **(A)** This plot depicts a low survival for postmenopausal samples compared to premenopausal women samples. **(B)** Cox-proportional hazard ratios plot showing significant variations between pre- and postmenopausal status. **(C)** This survival plot shows differential probabilities between different types of recurrent and nonrecurrent samples. **(D)** Cox-proportional hazard ratios plot showing significant variations between triple-negative, Her2-positive, and Her2-negative subtypes of breast cancer. **(E)** Survival plot for triple-negative, Her2-positive, and Her2-negative subtypes of breast cancer. **(F)** Survival plot for different stages of breast cancer. **(G)** The plot is for displaying different survival probabilities for samples belonging to different tumor grades.

The Kaplan–Meier plots depict survival for different clinical parameters of Indian breast cancer patients. Properties such as menopausal status, hormone receptor status, tumor grade, recurrence, and stages were analyzed among the cohort. There were 381 patients with data available for menopause status, and among them, there were two groups: pre (*n* = 216) and post (*n* = 159). A mildly significant (*p* = 0.11) low survival was observed for premenopausal patients when compared to postmenopausal patients (Figure 1A). A multivariate Cox proportional hazards analysis of the menopause status revealed that postmenopausal patients displayed a hazard ratio of 0.51, indicating that this group had half the risk of death when compared to premenopausal patients (Figure 1B). When the disease recurrence parameter was checked, the recurrent patients were divided into local, distant, distant + regional, and local + distant + regional based on where the recurrence occurred. When all these categories were compared, the local + distant + regional group had poor survival (*p*-value< 0.0001), followed by distant recurrence and local recurrence (Figure 1C). Breast cancer is classified commonly based on the expression of hormone receptors. Within the hormone receptors subtypes, Her2-positive subtype had worse prognosis (*p* = 0.026 < 0.05) (Figure 1E). A multivariate Cox proportional hazards analysis of the hormone receptor subtypes indicated that Her2-positive patients displayed a significant (*p*-value 0.097) hazard ratio of 2.4, indicating that this subtype has a high risk of death (Figure 1D). Among the different stages in our cohort, it was observed that stage IV exhibited worse survival than the others, with a significance of *p*-value < 0.0001 (Figure 1F). Patients falling in grades 1, 2, and 3 were plotted to analyze the survival based on the tumor grade. Patients with a higher grade had low survival when compared to grades 1 and 2 (p-value = 0.011) (Figure 1G). Among the 381 samples used for analysis, 17 matched tumor–normal samples were subjected to RNA sequencing analysis to identify DEGs and pathways regulated in the presence/absence of hormone and pre- and postmenopausal samples irrespective of hormone status.

### Gene expression and unique pathway alterations segregate six breast cancer subtypes

**FIGURE 2:**
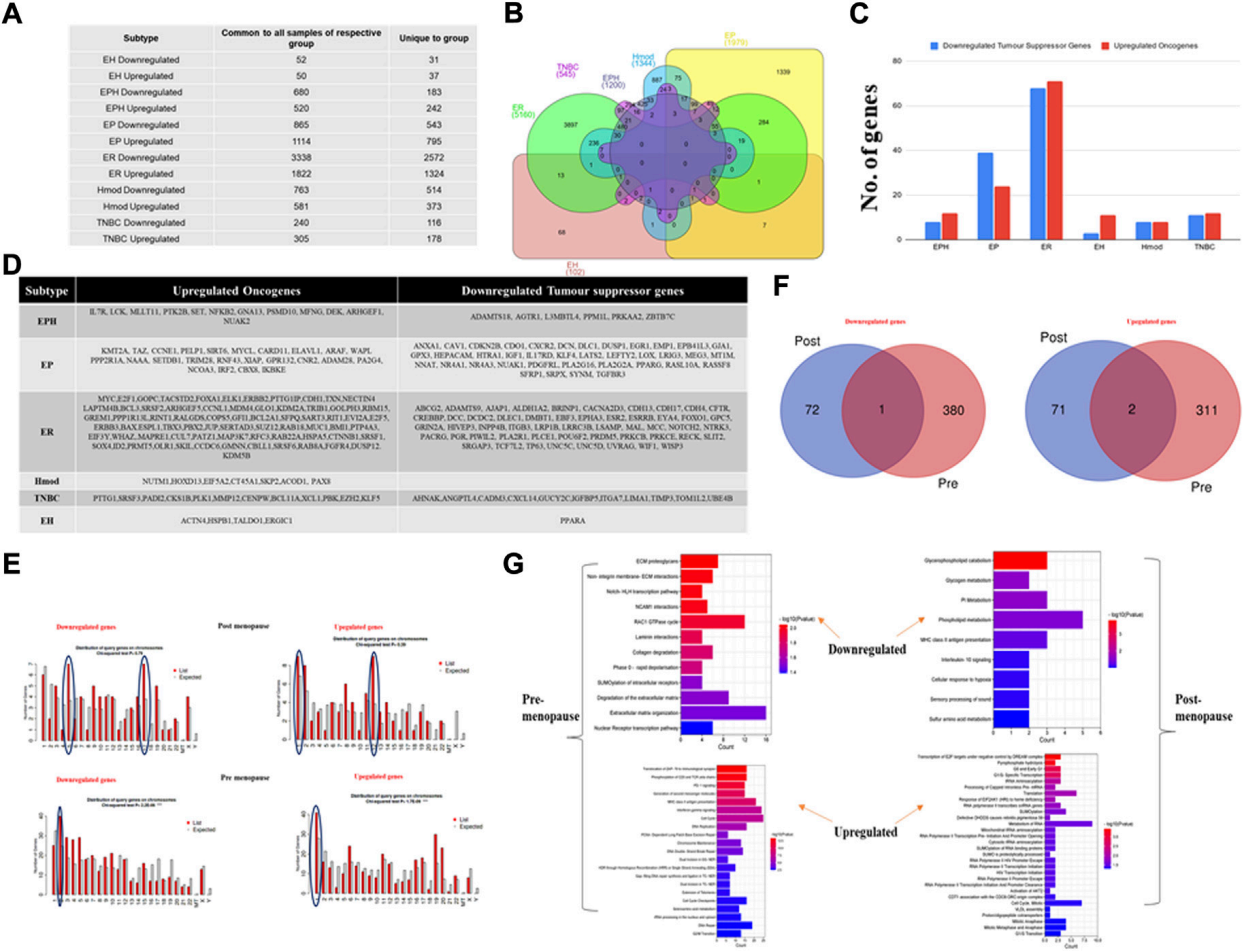
**(A)** Table showing the number of differentially expressed genes that are common to all patients in a subtype and unique genes when compared to other subtypes of breast cancer. **(B)** Venn diagram showing common and unique genes among different subtypes of breast cancer patients. **(C)** A bar graph depicting the number of upregulated oncogenes and downregulated tumor suppressor genes in six subtypes of Indian breast cancer patient samples. **(D)** A table with a list of upregulated oncogenes and downregulated tumor suppressor genes. **(E)** Venn diagram showing common and unique genes among pre- and postmenopausal Indian breast cancer patient samples. **(F)** Bar graphs depicting gene distribution on chromosomes in pre- and postmenopausal Indian breast cancer patient samples. **(G)** Bar graphs representing significantly upregulated and downregulated pathways in pre- and postmenopausal Indian breast cancer patient samples. The *y*-axis shows pathway terms and the *x*-axis is the gene count. The color gradient of the bar is based on the *p*-value.

A differential gene expression analysis was performed on tumor and matched-normal ER (3 pairs), EP (3 pairs), triple-positive (EPH, 4 pairs), Hmod (2 pairs), EH (2 pairs), and TNBC (3 pairs) patients. Among six subtypes, the ER subtype had a maximum alteration in gene expression where 2,572 genes were uniquely significantly downregulated, while 1,324 were upregulated (log2 fold change ≤ and ≥͢͢ 1) followed by EP (543 down and 795 up), Hmod (514, 373), EPH (183 and 243), and TNBC (116 and 173) and was the least in EH (31 and 37) (Figure 2A). As expected, a minimal overlap was observed between the subtypes, with ER having maximum overlap with EPH, EP, and EH (Figure 2B). It is well known that a balance tilt in oncogenic (ONC)/tumor suppressor (TSG) drives oncogenesis; we checked for alterations in ONC and TS across the subtypes. The DE genes were subjected to an oncogene/tumor suppressor analysis using breast cancer–specific oncogenes (https://oncovar.org/) and tumor suppressors (https://bioinfo.uth.edu/TSGene/). Each of the subtypes was analyzed for upregulated oncogenes and downregulated tumor suppressors. Most downregulated TSGs, and upregulated oncogenes, were observed in EH (16% TSG and 27% ONC), followed by TNBC (7.7% TSG and 5.6% ONC). The fewest alterations were observed in Hmod (0.97% TSG and 1.3% ONC), followed by EPH (4.37% TSG and 4.9% ONC), EP (6.9% TSG and 2.7% ONC), and ER (2.7% TSG and 5% ONC), indicating differences in the alterations in oncogenes and tumor suppressors among breast cancer subtypes (Figure 2C). Figure 2D depicts the list of significantly upregulated oncogenes and downregulated tumor suppressor genes in each subtype. Oncogenes such as MYC, SIRT6, IL7R, CCNE1, PAX8, and BCL11A were upregulated and TSGs DUSP1, AGTR1, NOTCH2, CREBBP, and ITGA7 were downregulated in the subtypes.

Furthermore, to identify the deregulated pathways, the upregulated and downregulated genes for each subtype were given as an input separately to the Reactome database, and the results were filtered for *p*-value < 0.01, and the pathways with a gene count of more than three were selected. The top results were represented in a bubble plot. Among the notably affected pathways were downregulated keratinization and RUNX3-related pathways among the ER samples; downregulated ubiquitination and upregulated FGFR signaling among Hmod; ECM interactions and notch signaling downregulated in TNBC; and upregulated collagen and cellular pathways. AP2-related genes were regulated in opposite directions in ER and Hmod (Supplementary Figures S1, S2). Ki67 is a well-known marker for tumor cell proliferation, therefore, based on the expression of Ki67, we classified Indian breast cancer patients into Ki67-high and Ki67-low groups and performed pathway analysis. Ki67-high patients displayed upregulation of matrix metalloprotease, platelet activation, and DNA methylation as the significant pathways. In Ki67-low patients, the noncanonical NF-kB pathway, interleukin signaling, and PI3k signaling were significantly upregulated suggesting that the observed pathways are independent of the cell cycle.

For the differences observed in survival between pre- and postmenopausal patients and understanding that premenopausal breast cancer is aggressive, we checked for pathways that regulate these phenotypes.

### Pre- and postmenopausal samples show unique pathway signatures

The breast cancer patient samples were divided into two categories, pre- and postmenopausal, based on the menopause data from the clinical features procured from the hospital. Genes with log2 fold change <1 and >−1 were filtered out for each patient. The common DEGs were pulled out from patients from each group and then further analyzed. Venn was performed to identify common and unique genes among the two types (Supplementary Material S1). Premenopausal samples showed 72 downregulated and 71 upregulated genes, whereas postmenopausal samples displayed 380 downregulated and 311 unique upregulated genes. Among the common genes analyzed, 1 was downregulated and 2 were upregulated (Figure 2F). These unique genes were then checked for chromosome distribution, and it was found that downregulated genes were on chromosomes 5, 17, and 2 in the post- and premenopausal samples, respectively (Figure 2E). The upregulated genes were primarily present on chromosome 1 for both post- and premenopausal samples, with chromosome 12 being additional for the postmenopausal samples. These unique significant upregulated and downregulated genes were given as input separately to the Reactome database to obtain deregulated pathways. In the samples of postmenopausal breast cancer women patients, the pathways related to metabolisms such as phospholipid metabolism, amino acid metabolism, and glycogen metabolism were downregulated, and the cell cycle processes connected to transcription and translation were upregulated. In the case of samples of premenopausal breast cancer women patients’, extracellular matrix regulation and collagen-dependent pathways were downregulated. Single- and double-stranded DNA repair and immune-related pathways were upregulated (Figure 2G), indicating deregulated cell cycle and metabolism as the reason for cancer progression in postmenopausal BC patients. By contrast, deregulated DNA damage and repair and altered immune signaling led to cancer progression in premenopausal BC patients.

Furthermore, to check whether RNA-seq can be used for subtyping breast cancer in the Indian cohort, using the existing PAM50, MammaPrint, and Oncotype DX, PCA was performed.

### 25-Gene set identified for Indian breast cancer cohort

**FIGURE 3:**
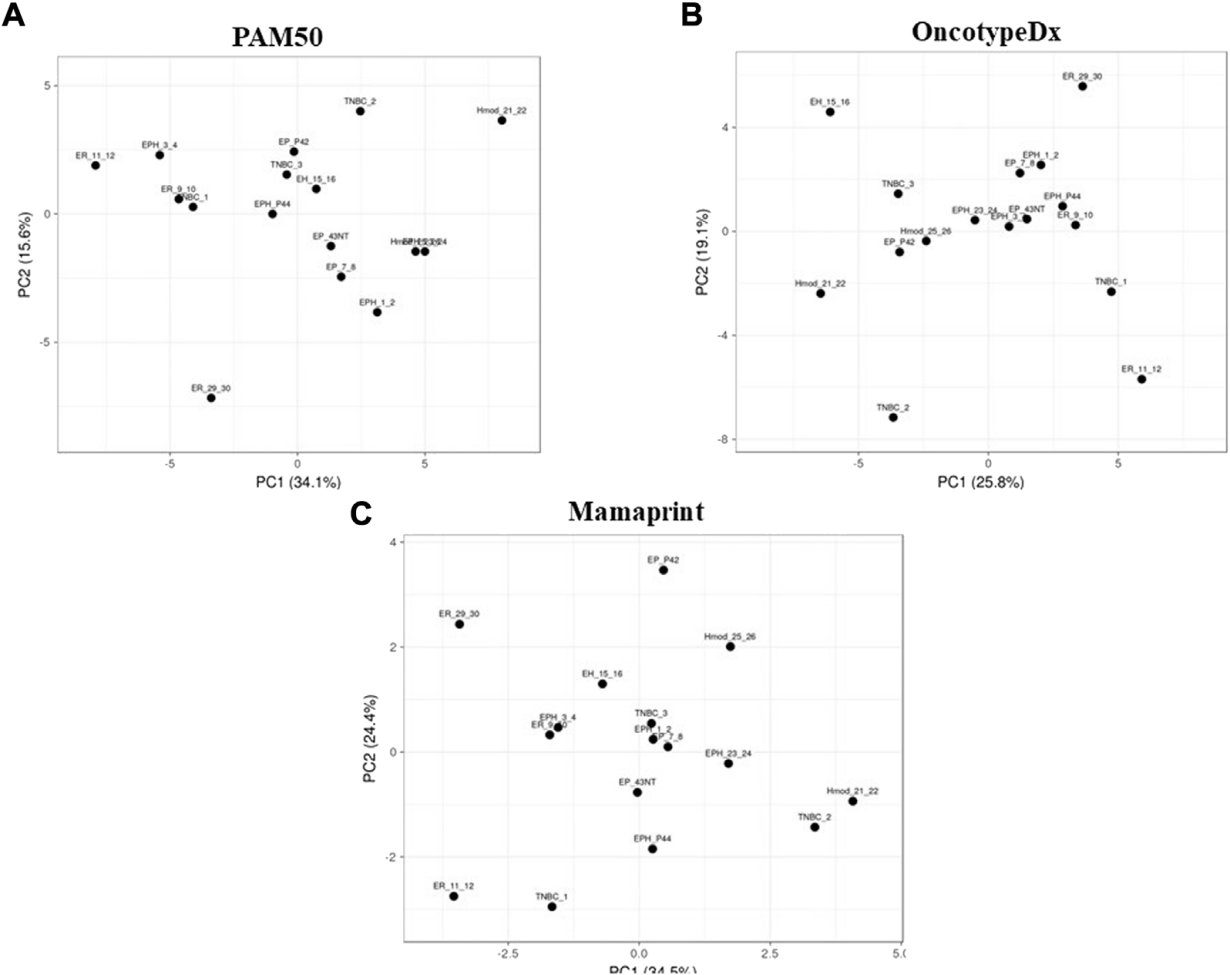
Principal component analysis of Indian breast cancer patient samples with the **(A)** PAM50, **(B)** MammaPrint, and **(C)** Oncotype DX gene sets.

Although gene expression patterns are unique for each subtype, no segregation was observed when the PCA was performed. We performed PCA using gene sets of PAM50, MammaPrint, and Oncotype DX. We did not observe clear segregation of the subtypes in the Indian cohort, possibly due to differences in the microarray and the RNA-DESeq–based analysis (Figures 3A–C). To check if the absence of segregation can be due to differences in the technology used, we downloaded RNA-seq data from TCGA and analyzed for these gene sets; clear segregation was observed among hormone-positive and hormone-negative samples (Supplementary Figure S3A–C) in the PCA, suggesting that it was not dependent on the technology used. The samples from TCGA mainly belong to the Caucasian population, showing a distinct separation. The panels are designed primarily for a specific population, suggesting population-specific expressions that may underlie observed differences.

**FIGURE 4:**
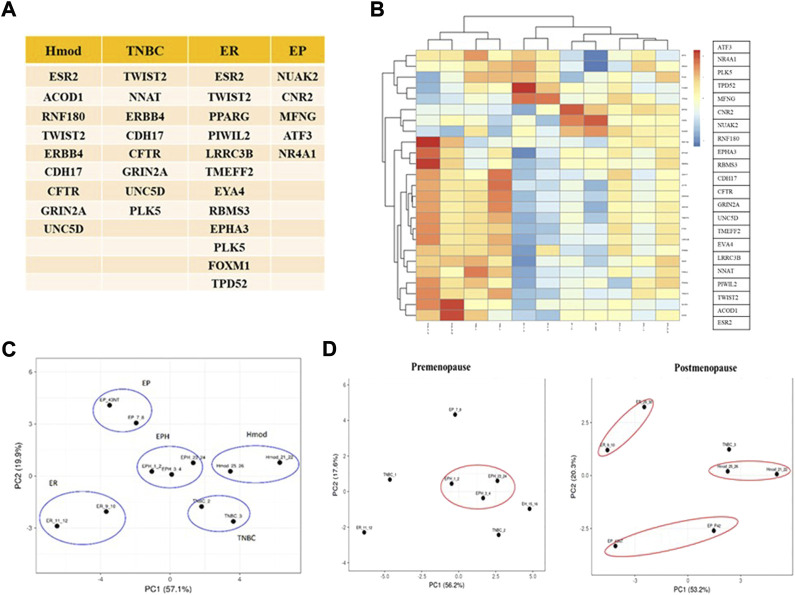
**(A)** Table depicting unique mRNAs with potential as Indian-specific biomarkers derived from different subtypes. **(B)** Heat map of unique mRNAs chosen as potential biomarkers in the Indian population. Blue represents downregulated genes and red represents upregulation. **(C)** PCA plot showing segregation of Indian patients with selected mRNAs. **(D)** PCA plots showing segregation with selected mRNAs between pre- and postmenopausal Indian breast cancer patients.

Because no clear segregation of the BC subtypes in the Indian cohort was obtained with existing panels, and to narrow the gene set down that might segregate the subtypes, the list of genes based on log2 fold change, *p*-value, and a significant DEG list for each patient was obtained. For each subtype, significant common genes were obtained by comparing all the patients belonging to that subtype. This set of genes was then compared among the subtypes, and a unique DEG list was obtained for each subtype. Among the unique DEG list, the genes already known in the literature relevant to cancer were narrowed down. The PCA and heat maps were iteratively used to narrow these lists into combinations that segregated the patient samples into their different hormone receptor–based subtypes. Twenty-five mRNAs were identified specific to our data (Figures 4A,B). The selected mRNAs showed proper segregation in the PCA of hormone subtypes in the Indian cohort (Figure 4C). Also, the candidate gene set was used to evaluate segregation between pre- and postmenopausal women samples in the Indian cohort. Postmenopausal breast cancer samples showed better segregation in the PCA of the subtypes than did the premenopausal samples (Figure 4D). To check whether the 25-gene sets could segregate BC subtypes of the TCGA cohort, RSEM-normalized values for 946 individuals were downloaded from TCGA. The samples were segregated into pre- and postmenopausal PCA plots for a 25-gene set plotted for the TCGA samples. Surprisingly, we observed that premenopausal samples showed better segregation than did postmenopausal samples. The existing gene sets (PAM50, MammaPrint, and Oncotype DX) could only segregate postmenopausal TCGA samples. Hence, the 25-gene set could be used for segregating the BC subtypes of premenopausal women in the Caucasian population (Supplementary Figure S3D). The genes responsible for differential segregation of the pre- and postmenopausal BC patients in the Indian cohort and the TCGA were dependent on differential expression of genes such as CNR2 (Luminal B), LRRC3B, EYA4, TMEFF2 (Luminal A), ESR2, GRIN2A, ERBB4, and NNAT (ER−ve aka Hmod and TNBC). These are, therefore, of particular interest as population-specific markers.

Since we did not find segregation of the premenopausal samples and to check if adding lncRNA to the panel improves segregation of the breast cancer subtypes, we performed a differential lncRNA analysis across subtypes.

### Unique lncRNA expression pattern in Indian breast cancer subtypes

**FIGURE 5:**
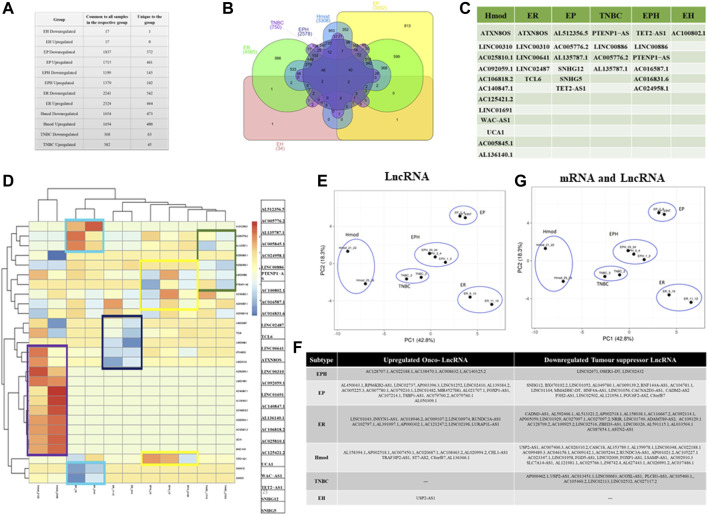
**(A)** Table showing the number of differentially expressed lncRNAs that are common to all patients in a subtype and unique lncRNAs when compared to other subtypes of breast cancer. **(B)** Venn diagram showing common and unique lncRNAs among different subtypes of breast cancer patients. **(C)** Table depicting unique lncRNAs with potential as Indian-specific biomarkers derived from different subtypes. **(D)** Heat map of unique lncRNAs chosen as potential biomarkers in the Indian population. Blue represents downregulated genes and red represents upregulation. **(E)** PCA plot showing segregation of Indian patients with selected lncRNAs. **(F)** Table showing onco and tumor suppressor lncRNAs segregated subtype wise. **(G)** PCA plot of combined signature of mRNA and lncRNA for Indian breast cancer patient samples.

LncRNA regulates gene expression and is known for its tissue-specific expression (Jiang et al., 2016; Perron et al., 2017). To identify subtype-specific lncRNA, DESeq was performed using matched normal/tumor pairs for each sample, and lncRNAs which were either upregulated or downregulated in all samples of a group were obtained. Among the subtypes, ER showed the most significant number of alterations in lncRNA as was observed for mRNA, followed by Hmod and EP, and the least in EH. Triple-negative and triple-positive cancer showed comparable alterations in both upregulated and downregulated lncRNAs (Figure 5A). To obtain commonly regulated differential lncRNAs, Venn was used. No common lncRNA to all subtypes was observed (Figure 5B), indicating subtype specificity of lncRNAs. TRG-AS1, MAFA-AS1, and MELTF-AS1 in EPH; TET-AS1, ZNF26-DT, and C4A-AS1 in TNBC; FZD4-AS1, CHL1-AS1, and B4GALT1-AS1 in Hmod; HOTAIR, EGOT, FOXN3-AS2, and TMEM12-AS1 in ER; DOCK9-AS1, MORC1-AS1, and GASAL1 in EP were some of the uniquely upregulated lncRNAs in a subtype-specific manner. ARNTl2-AS1, ELMO-AS1, and NAMA in EPH; B4GALT1-AS1, HOXB-AS1, and EP300-AS1 in TNBC; NCF4-AS1, ZSWIM8-AS1, and DICER1-AS1 in Hood; NRIR, TP53TG1, and DDX11-AS1 in ER; MYLK-AS1, ADNP-AS1, SNHG12, and HNF4A-AS1 in EP (Supplementary Material S2) were some of the uniquely downregulated lncRNAs in subtypes.

To identify DE lncRNA between pre- and postmenopausal samples, DE lncRNA was obtained from the pre- and postmenopausal samples. Shared long intergenic noncoding RNA (lincRNA) and unique lncRNAs to pre- and postmenopausal patients were obtained. AL357054.2 was the only lncRNA commonly upregulated in the postmenopausal samples. LINC02306, AL442163.1, AC124947.1, and AC016831.1 were commonly downregulated, while AC024958.1 and AC011447.3 were commonly upregulated in premenopausal samples (Supplementary Material S1).

As in mRNA analysis, the oncogenes and tumor suppressors regulate tumorigenesis; we also classified the lncRNA as ONC and TSG and identified subtype-specific lncRNA (Figure 5F). The unique lncRNAs were analyzed for each subtype and were compared against the Lnc2cancer database, and the known breast cancer-related lncRNAs were selected. A set of 27 lncRNAs was identified from the data (Figure 5C). This gene set was devised iteratively following the removal of frequent outliers. It was observed that most lncRNAs were upregulated in Hmod, whereas the same had negligible expressions in all other subtypes.

Similarly, ER showed downregulated lncRNAs, which were upregulated in other subtypes. The expression pattern using lncRNA showed an apparent demarcation among the subtypes, as shown in the heat map (Figure 5D). Figure 5C shows that ATXN8OS, UCA1, SNHG12, SNHG5, LINC02487, TCL6, TET2-AS1, and PTENP1-AS were the identified lncRNA sets from our data. The selected LncRNA segregated different breast cancer subtypes in our cohort (Figure 5E). When the same lncRNA set was compared in pre- and postmenopausal women samples, Hmod and ER subtypes segregated better in the postmenopausal sample, and premenopausal samples did not show any clear pattern in the PCA (Supplementary Figure S3E). Since lncRNA signatures also segregated the subtypes only in postmenopausal samples, we combined the mRNA and lncRNA list and checked for the segregation of subtypes of BC.

### 25 mRNA and 27 lncRNA signatures segregate breast cancer subtypes in Indian cohort

The PCA of the patients shows an immediate improvement over the existing standard gene sets (PAM50, MammaPrint, and Oncotype DX) in the segregation of hormone receptor subtypes in the PCA with mRNA and lncRNA signature from our data. The clear separation of Hmod (moderate Her2 expression, ER/PR negative) from the other subtypes is noted, as is also visible in the heat map of lncRNAs. These are particularly interesting as they are Her2-specific lncRNAs (Figure 5G). Furthermore, the triple-negative (TNBC) and triple-positive (EPH) subtypes surprisingly cluster close together. The two luminal A groups, ER and EP, do not cluster closely, indicating the heterogeneity observed within luminal A tumors. When the combined list was checked for pre- and postmenopausal samples, the pattern observed for only the previous lncRNA signature list repeated as Hmod and ER was seen as a distinct cluster in the postmenopausal samples, and an improvement from the previous signatures was observed in the premenopausal samples where the EPH subtype segregated from other subtypes (Supplementary Figure S3F) in the PCA.

Furthermore, to understand if the mRNA-lncRNA signatures could also have prognostic value, we performed LASSO-Cox. We selected luminal subtype signatures obtained from the Indian cohort and validated the performance of the signatures in luminal A subtype from the TCGA data set.

### Combined mRNA and lncRNA signature predicts survival in luminal A breast cancer subtype in TCGA data set

We performed LASSO-Cox using 25 genes obtained as the signature from the Indian cohort analysis to check its prognostic performance. Only six mRNAs showed mild correlation with survival. When the analysis was performed with lncRNAs obtained from the Indian cohort and tested in the TCGA cohort, no lncRNA showed correlation with survival. We checked for lncRNA correlation in other subtypes of breast cancer. We observed lncRNA TCL6 associated with survival in Her2+ve cancers in the TCGA cohort with a CI of 0.86 (Supplementary Figures S4Ai, ii). Furthermore, we merged the gene list of lncRNAs and mRNAs and performed LASSO (Supplementary Figures S4Bi), and we obtained three gene signatures which performed better than the mRNAs alone in prediction of survival. We also performed univariate and multivariate Cox analyses and identified three gene signatures. The three gene signatures consisted of LRRC3B, GRIN2A, and SNHG12. To check whether the three genes' performance in predicting survival was significant, we used the risk score of genes and lncRNAs (Supplementary Figure S4Bii) and categorized the patients into two groups of low risk and high risk and performed a survival analysis using the KM plot (*p*-value −0.0093) (Supplementary Figure S4Biii), suggesting the prognostic performance of the combined lncRNA and gene. Also, when these three genes were checked for interactions with chemotherapeutic drugs in DGIdb (Cotto et al., 2018), an interaction score of 1.37 was returned for GRIN2A with the drug dizocilpine (North et al., 2010).

Having identified the lncRNAs specific to each subtype and their added prognostic value, we checked for the lncRNA-mRNA pairs that were co-expressed in all subtypes of breast cancer to understand the functional significance of the lncRNA in breast cancer pathogenesis.

### Unique lncRNA-mRNA signature in breast cancer subtypes

**FIGURE 6:**
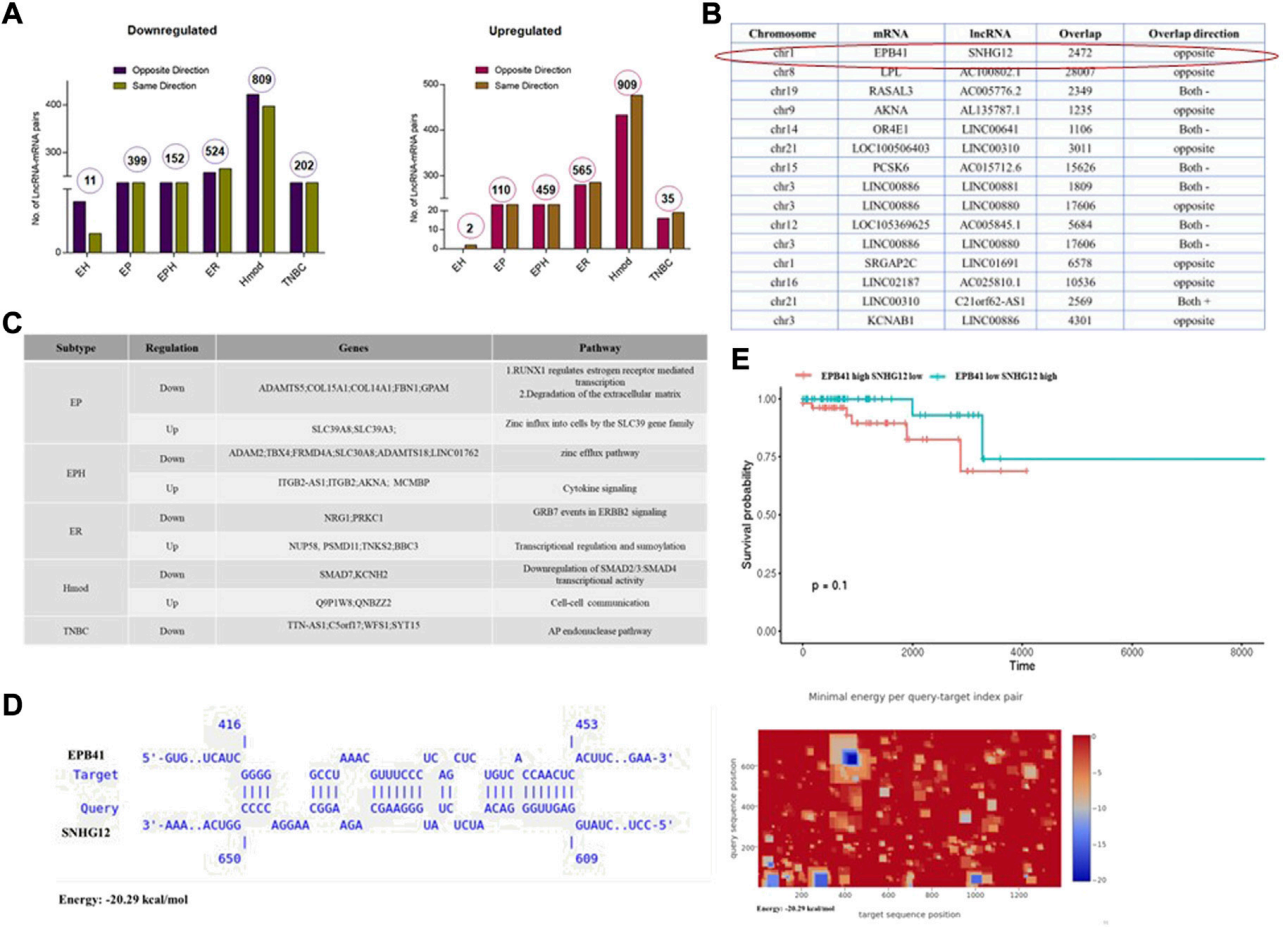
**(A)** Bar graphs depicting number of lncRNA-mRNA pairs in different subtypes of Indian breast cancer patient samples. **(B)** Table shows lncRNA and its corresponding mRNA pair obtained from different subtypes that are common to all the patients in the group. **(C)** Table depicting subtype-specific pathways obtained from lncRNA-mRNA pairs. **(D)** A potential binding site between EPB41 (Target) and SNHG12 (Query) was identified by IntaRNA. The heat map shows potential binding sites between EPB41 and SNHG12 in blue. **(E)** Survival plot for high and low SNHG12 and EPB41 levels in the TCGA data set.

To identify potential functions of the lncRNAs, we identified potential *cis*-acting lncRNA-mRNA pairs on the basis of their overlap on the chromosomes. Although lncRNA regulates gene expression in *cis* and *trans*, we focused on the lncRNA-mRNA pairs in *cis* with an overlap of 1,000 bp. Hmod showed a maximum number of *cis*-acting lncRNA-mRNA pairs (809 downregulated) (909 upregulated), followed by ER (524 downregulated and 565 upregulated), which was in contrast to mRNA expression alone. The gene-lncRNA pair found in the same orientation (5′-3′-5′-3′) *vs*. opposite orientation (5′-3′-3′-5′) is presented as a bar graph in Figure 6A. We performed a Pearson correlation to correlate the overall expression of mRNA and lncRNA in a subtype-specific manner. We found a minimal correlation in ER (r = 0.15, p = 8e−11), and other subtypes had no significant correlation. Interestingly, 91% correlation in the EPH subtype was observed when a Pearson correlation analysis was performed using downregulated and upregulated *cis* lncRNA-mRNA pairs separately. All other subtypes did not show a significant correlation.

Furthermore, subtype-specific lncRNA-mRNA pairs with a Pearson correlation of at least 90% were segregated (Figure 6B). The lncRNA was checked in the TANRIC database for expression status and subtype specificity. Subtype-specific differences were observed in WAS-AS1, expressed highly in basal in the TCGA. By contrast, it is specific to Hmod and was not observed in basal in the Indian cohort. SNHG12 is highly basal in the TCGA data sets, whereas it is downregulated in EP in the Indian cohort. Linc00861 showed a downregulated expression pattern in both the TCGA and Indian cohort, whereas SLC39A8 was high in the TCGA data and EP subtype in the Indian cohort and was associated with better survival.

The genes-lncRNA pair from each subtype was subjected to pathway analysis, and unique pathways were regulated in each subtype (Figures 6A,C). The downregulated pathways were zinc efflux transporters in EPH, whereas zinc influx was upregulated in the EP subtype. Some of the underrepresented subtypes in mRNA were observed when the lncRNA-mRNA analysis was carried out. A combined analysis of lncRNA-mRNA returned some of the critical players in oncogenesis. To find out the lncRNA regulation of mRNA, several tools are available which can be used to identify the mode of action of lncRNA. We had noted that TSG SNHG12 was downregulated, and the *cis* gene ONC EPB41 was upregulated; we sought to narrow down the mechanism using *in silico* methods.

### SNHG12 may regulate EPB41 specific to the EP subtype

To identify potential functions of the lncRNAs, potential *cis*-acting lncRNA-mRNA pairs were identified on the basis of the overlap on the chromosomes. Among the lncRNAs, SNHG12 is oncogenic and participates in proliferation, invasion, and metastasis in breast cancer tumors (Wang et al., 2017; Tamang et al., 2019; Zimta et al., 2020). In our cohort, SNHG12 was deregulated in the EP subtype. This lncRNA was picked up and its mRNA pairs identified (Figure 6B). Erythrocyte membrane protein band 4.1 (EPB41) was one of the interesting targets as it is known to play a role in the invasion of other cancers (Yang et al., 2016; Zhao et al., 2020; Yuan et al., 2021). We wanted to see its binding and interaction with SNHG12.

The IntaRNA tool was used to check for the binding between lncRNA SNHG12 and EPB41. The results indicate a feasible binding between the two (Figure 6D). While various regulatory functions of SNHG12 and EPB41 have been elucidated, the potential interaction between them remains unexplored and is a potential direction for further research. Similarly, to find potential proteins that can bind to SNHG12 RNA, the eCLIP-validated proteins were collated from the RNAct database (Lang et al., 2019) and checked for possible loss of oncogenic protein/PRC binding, which would block oncogene expression. We found 137 proteins that could bind to SNHG12 from the RNAct database, of which 5 (GTF2F1, APOBEC3C, DKC1, SUGP2, and TIA1) were present in the gene list common to all EP patients.

One of the hallmarks of cancer is evasion of immune response, and EPB41, a cytoskeletal protein, has a role in dendritic cell synapse and its role in the immune system involves antigen presentation (Zhao et al., 2020). EPB41 has also been shown to increase cell proliferation and invasion (Zeng et al., 2016). SNHG12 may have a role in polarization of immune cells, providing advantage for cancer cell growth (Tamang et al., 2019, 12). Therefore, we checked for any association that SNHG12 and EPB41 may have with immune cell function.

### SNHG12 and EPB41 expression status may regulate immune cell function

SNHG12 showed significant association with survival (HR: *p*-value < 0.064) in luminal A subtype of breast cancer in the TCGA cohort, while EPB41 had no correlation with survival. Since we hypothesized that these genes might be co-expressed and have a combined effect on survival, we performed the survival analysis with EPB41 high and SNHG12 low *vs*. EPB41 low and SNHG12 high conditions, and we found an association with survival at *p*-value-0.1) (Figure 6E). Furthermore, to test whether the change in survival might have an association with the immune cell status, we performed a CIBERSORT analysis (Chen et al., 2018). We found that SNHG12 low and EPB41 high had relatively low macrophages, high Tregs, and plasma cells (Supplementary Figure 4C), which might be associated with poor survival. Furthermore, we also checked the status of immune cells in normal breast tissue and found that in SNHG12 low and EPB41 high, Tregs was significantly high (*p*-value-0.00000000666), and dendritic cells (*p*-value-0.01451) and plasma cells (*p*-value-0.00000000000221) among other immune cells were significantly higher indicating further that the genes might be involved in polarization of the immune cells which might contribute to the differences in survival.

### Validation of known cancer genes in Indian breast cancer patients

**FIGURE 7:**
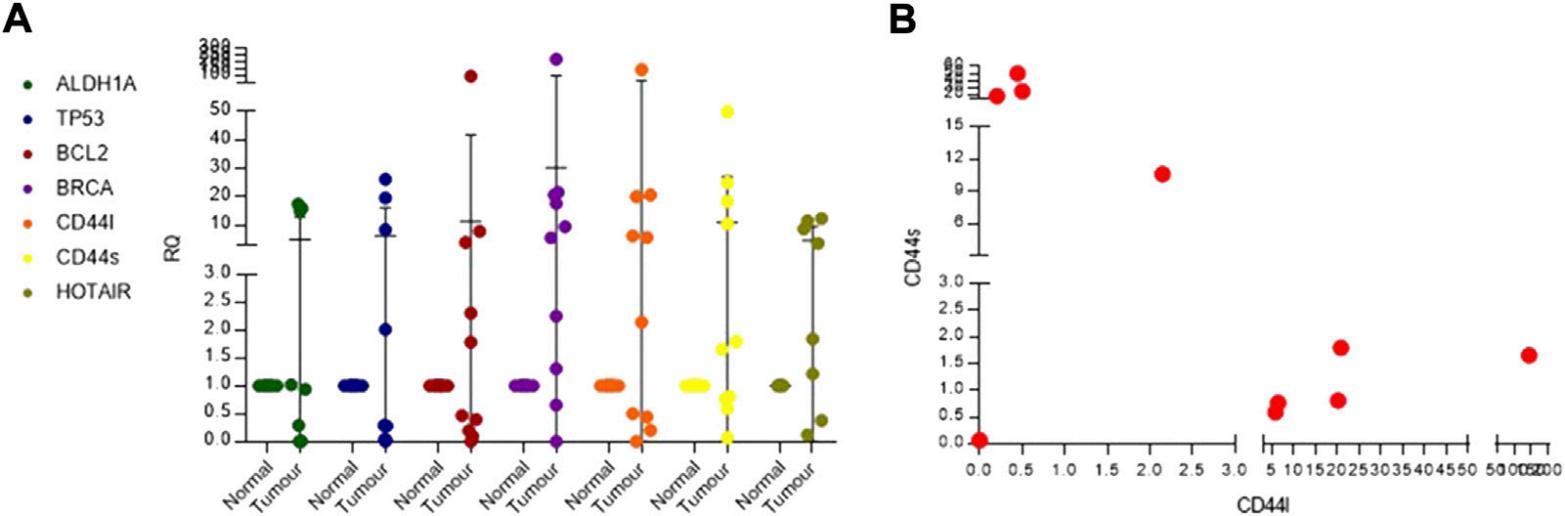
**(A)** Real-time PCR dot plot depicting relative quantification for known breast cancer genes in Indian breast cancer patients. **(B)** Scatter plot for checking co-expression of CD44l and s forms.

We selected five breast cancer–relevant genes, namely, ALDH1A, BRCA, TP53, BCL2, and CD44, for validation, using SYBR Green real-time PCR assays in *n* = 10 IDC samples. We observed that 40% of patients showed upregulation of ALDH1A and TP53. BCL2, an anti-apoptotic gene, was overexpressed in 50% of the patients. BRCA1 was commonly seen upregulated in 80% of the patients (Figure 7A). CD44 long- and short-form levels were checked. It was observed that 60% of the patients showed upregulation of CD44 long and short (CD44l and s) forms. Commonly deregulated lncRNA in breast cancer HOTAIR levels were also checked, and it was observed that 77% of the patients showed a higher expression than the normal samples. When the patients were analyzed for CD44l and s forms separately, it was seen that 50% of the patients had high levels of CD44l form and low levels of CD44s form, and 30% of the patients had high levels of CD44s form and low levels of l form (Figure 7B).

## Conclusion

A transcriptome sequencing and analysis of 17 Indian breast cancer tumors and matched normal showed that already existing microarray gene signatures failed to segregate the samples into their subtypes using the PCA. Every subtype showed a unique gene and pathway signature with minimum overlap. A unique set of DE onco and tumor suppressor lncRNA was identified for each subtype. Our data identified an mRNA-lncRNA gene set that could segregate pre- and postmenopausal women with breast cancer. This is the first study reporting subtype-specific mRNA and lncRNA expression in Indian breast cancer patients. However, all these results need validation with a bigger sample size.

## Discussion

Breast cancer is heterogenous and one of the major causes of death in women worldwide (Manjunath and Choudhary, 2021). Better insight into the molecular basis of this is possible when new approaches like next-generation sequencing are used (Casamassimi et al., 2017). Most of the breast cancer data available in the repositories are from the Caucasian population (Bhattacharyya et al., 2020). The gene signatures already available are from this population, and population-specific changes are not very well addressed. Therefore, region-specific data generation with subtype information is necessary. One of the aims of our study was to generate breast cancer patient data for the Indian population categorized into six different subtypes based on the hormone receptor status and to check for subtype-specific gene and lncRNA signatures. Our RNA-seq data from 17 samples showed subtype-specific changes. The number of samples sequenced is a limitation of this study. However, the results obtained could be further validated in the larger data set. Maximum alteration was observed in ER with 2,572 downregulated genes and 1,324 upregulated genes followed by EP, Hmod, EPH, TNBC, and finally EH. Among the deregulated pathways, ER-positive subtypes showed keratinization, and RUNX3 and AP2 family of genes regulating transcription and metabolism pathways. ER-negative tumors showed deregulation of ubiquitination, FGFR signaling, ECM interactions and notch signaling, and collagen and cellular pathways. Very few gene expression studies have been reported from India to date. One of the very early studies by Thakkar et al. (2010) showed 108 DEGs in 31 ER-positive breast tumors using microarray analysis. They found that these genes were mostly involved in mRNA transcription and cellular differentiation pathways. Another study also used microarray technology and sequenced 29 tumors categorized into luminal, basal, and Her2, and 9 normal samples. They showed cell cycle, DNA replication, lipid metabolism PPAR signaling, focal adhesion, and metastasis to be deregulated in Indian samples (Malvia et al., 2019). Furthermore, pathways related to collagen, focal adhesion, and ECM were reported to be deregulated in various cancers such as breast tumors in other populations (Bergamaschi et al., 2008; Lee et al., 2008; Luo and Guan, 2010; Oskarsson, 2013; Acerbi et al., 2015; Insua-Rodríguez and Oskarsson, 2016).

lncRNAs are a class of noncoding RNAs with lengths between 200 and 200,000 bases (Huarte, 2015). They lack protein-coding features such as open-reading frames. They bear many similarities to mRNAs, often having multiple exons and undergoing posttranscriptional changes such as splicing, polyadenylation, and 5′-capping (Prensner and Chinnaiyan, 2011). In several cases, the dysregulation of lncRNAs has been found to be directly or indirectly associated with the hallmarks of cancers, mediated by other interacting partners such as proteins, other noncoding RNAs, transcription factors, and histone complexes (Zimta et al., 2020). Studies done previously from the western population have shown HOTAIR lncRNA to be overexpressed in HER2+ breast cancers and HOTAIRM1 in basal-like breast cancers (Su et al., 2014). LINC160 and DSCAM-AS1 were seen to be highly expressed in luminal A and B, respectively (Jonsson et al., 2015; Vu et al., 2016). H19, MALAT, BC200, XIST, and ATB are the other lncRNAs frequently deregulated in breast cancer (Iacoangeli et al., 2004; Sirchia et al., 2009; Hansji et al., 2014; Kim et al., 2018, 1). However, there is a dearth of explicitly Indian population-specific research evaluating lncRNAs in breast cancer. We analyzed our sequenced data for lncRNAs and found uniqueness in DE lncRNA in different subtypes. The ER subtype had the highest alterations in lncRNA followed by Hmod, EP, and EH. TNBC and triple-positive (EPH) cancer showed comparable levels of DE lncRNAs. ATXN8OS, UCA1, SNHG12, SNHG5, LINC02487, TCL6, TET2-AS1, PTENP1-AS were some of the unique lncRNAs found in our cohort from different subtypes that were deregulated. Another study on Indian breast cancer showed ADAMTS9-AS2, EPB41L4A-AS1, WDFY3-AS2, RP11-295M3.4, RP11-161M6.2, RP11-490M8.1, CTB-92J24.3, and FAM83H-AS1 to be deregulated in early-stage breast cancer (Deva Magendhra Rao et al., 2019). Among the DE lncRNAs in our data, SNHG12 (small nucleolar host gene 12), a lncRNA present on chromosome 1 at the p35.3 region, was looked into further. The length of SNHG12 is ∼1,867 bases coding for SNORA16A, SNORA61, SNORA66, and SNORD99 (Zhai et al., 2015; Lan et al., 2017, 12). SNHG12 has been implicated in various cancers, such as gastric cancer, triple-negative breast cancer, glioma, and osteosarcoma. In triple-negative breast cancer, gastric cancer, and glioma, SNHG12 is high in expression (Lan et al., 2017; Wang et al., 2017; Zhang and Lu, 2018, 12; Zhou et al., 2018, 12; Tamang et al., 2019, 12). ER-positive breast tumors in the TCGA data showed low expression of SNHG12 that correlated with our studies (Li et al., 2015). This also indicates tumor- and subtype-specific expression of SNHG12. In our data, SNHG12 was downregulated in the EP subtype hinting at a possible dual role as both oncogene and tumor suppressor which needs to be further investigated. Through eCLIP data from the RNAct database (Lang et al., 2019), we found proteins that could bind to SNHG12, and among them, GTF2F1, APOBEC3C, DKC1, SUGP2, and TIA1 genes were found in our list for the EP subtype. The role of the immune system in cancer is well established (Loose and Van de Wiele, 2009). Immune escape by the tumor is promoted by activation of tumor microenvironment features such as tumor-associated macrophages (TAMs), abnormal antitumor immune cells such as dendritic cells, natural killer cells, and regulatory T cells (Togashi et al., 2019; Wylie et al., 2019). Various lncRNAs are known to participate in interactions between a cancer cell and immune cells (Pi et al., 2021). Small nucleolar RNA host gene (SNHG) family members are known to regulate the biological function of immune cells. SNHG1, SNHG12, and SNHG16 regulate Treg cells and promote immune escape (Pei et al., 2018, 1; Tamang et al., 2019, 12; Ni et al., 2020, 1; Pi et al., 2021). Blocking SNHG12 might cause depolarization of refractory immune cells that are primed by tumor in non–small-cell lung cancer (Huang et al., 2022, 12). SNHG12 is known to promote immune escape in ovarian cancer cells (Qian et al., 2020, 12). EPB41 gene expression silencing has been known to elevate cell surface antigen in dendritic cells (Zhao et al., 2020).

From the clinical analysis of our data, recurrent samples and grade 3 and stage 4 samples showed poor survival that correlated with the other population data. Her2-positive cancers showed poor survival in our data. A study from India with 3,453 patients showed a 5-year overall survival to be 96.11% (95.12–97.1) in hormone receptor–positive/HER2-negative, 92.74% (90.73–94.8) in TNBC, and 90.62% (88.17–93.15) in HER2 subgroups (Doval et al., 2020). However, in a study conducted by Pan et al. (2020), with Asian breast tumors, Her2-positive cancers with an enriched immune score showed better survival. Low-grade HER2-positive breast cancer patients showed poor survival outcomes in European populations (Tovey et al., 2009).

Our RNA-seq data failed to segregate PCA PAM50, Oncotype DX, and MammaPrint. However, when we separated pre- and postmenopausal samples, we could see minimum segregation. DNA microarray data from Indian breast cancers had shown segregation for the PAM50 gene set in the study by Malvia S et al. Multiple breast cancer patient RNA-seq studies involving western populations have shown segregation for PAM50 gene set. A 25-mRNA and 27-lncRNA gene set was derived from our data after iteratively performing segregation. There are multiple studies available from the western population having gene signatures for breast cancer (Lee et al., 2008; Rathnagiriswaran et al., 2010; Arranz et al., 2012; Nielsen et al., 2014, 50; Dieci et al., 2016, 50; Kothari et al., 2020) but none for the Indian population. The limitation of this study is the sample size. Nevertheless, it is the only study that shows an mRNA-lncRNA gene signature for the Indian population that is subtype specific. This definitely shows some potential and a foundation for further studies. A larger sample size for sequencing and validation could be utilized next to strengthen the signatures obtained.

## Data Availability

The original contributions presented in the study are publicly available. This data can be found in NCBI, under accession number PRJNA835602.

## Abbreviations

RNA, ribonucleic acid; mRNA, messenger RNA; lncRNA, long noncoding RNA; BC, breast cancer; IDC, invasive ductal carcinoma; TNBC, triple-negative breast cancer; ER, estrogen receptor; PR, progesterone receptor; HER2, human epidermal growth factor receptor; TCGA, The Cancer Genome Atlas; PAM50, prediction analysis of microarray 50; FFPE, formalin-fixed paraffin-embedded; GLOBOCAN, Global Cancer Observatory: CANCER TODAY; ROR, risk of recurrence score; UCSC, University of California, Santa Cruz; SAM, sequence alignment map; BAM, binary alignment map; PCA, principal component analysis; NCBI, National Center for Biotechnology Information; ONC, oncogenes; TSG, tumor suppressor genes; DEGs, differentially expressed genes.
