# Supplementary material for: Transcriptomic profiling of Indian breast cancer patients revealed subtype-specific mRNA and lncRNA signatures
Source: Front Genet. 2022 Oct 25;13:932060. doi: 10.3389/fgene.2022.932060 (PMC9641000; doi:10.3389/fgene.2022.932060)
Supplement: Supplementary file 4 [file DataSheet1.PDF]

Figure 1

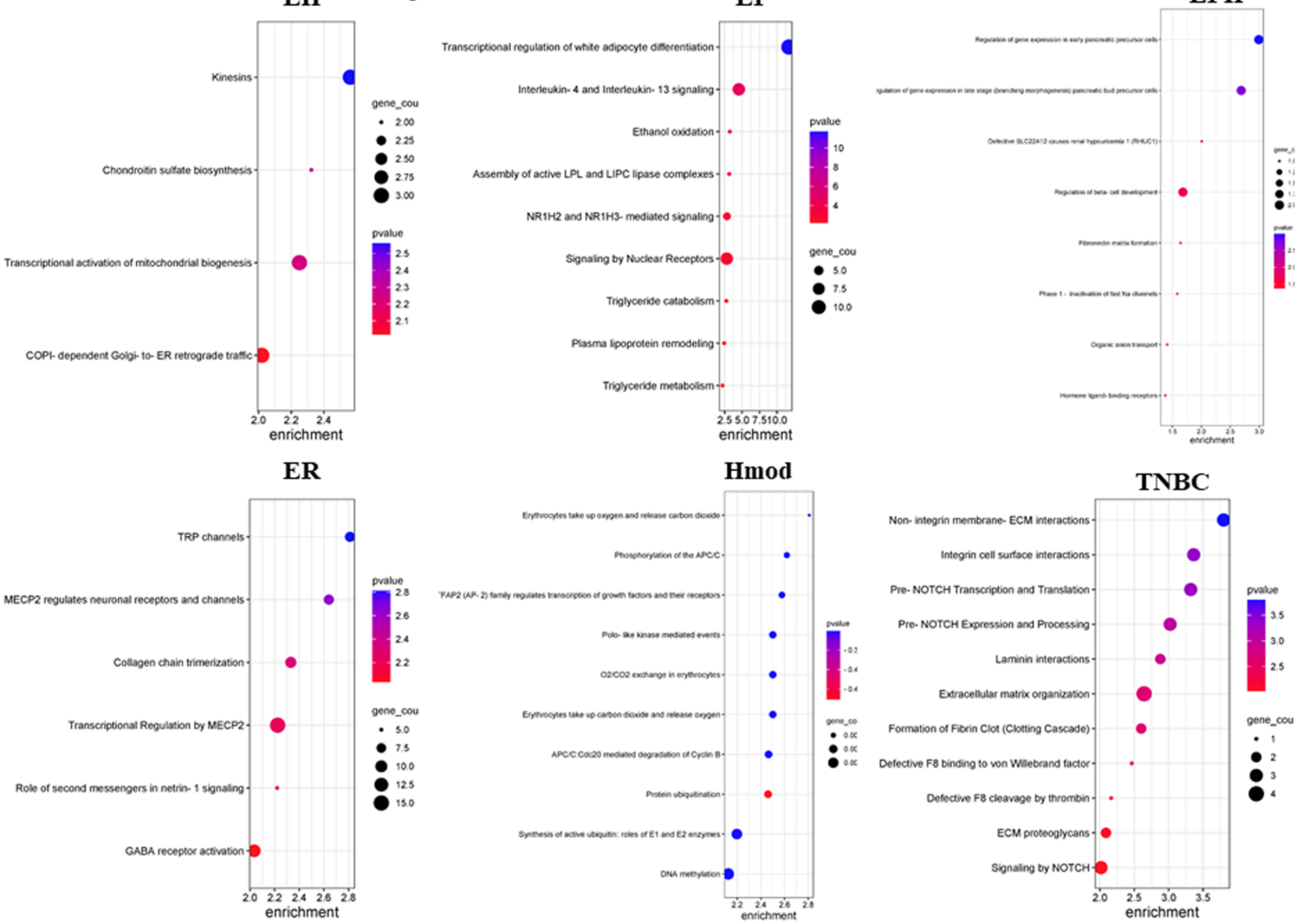
EH
Kinesins
Chondroitin sulfate biosynthesis
Transcriptional activation of mitochondrial biogenesis
COPI- dependent Golgi- to- ER retrograde traffic
2.0 2.2 2.4
enrichment
gene_cou
2.00
2.25
2.50
2.75
3.00
pvalue
2.5
2.4
2.3
2.2
2.1
EP
Transcriptional regulation of white adipocyte differentiation
Interleukin- 4 and Interleukin- 13 signaling
Ethanol oxidation
Assembly of active LPL and LIPC lipase complexes
NR1H2 and NR1H3- mediated signaling
Signaling by Nuclear Receptors
Triglyceride catabolism
Plasma lipoprotein remodeling
Triglyceride metabolism
2.5 5.0 7.510.0
enrichment
pvalue
10
8
6
4
gene_cou
5.0
7.5
10.0
EPH
Regulation of gene expression in early pancreatic precursor cells
gulation of gene expression in late stage (branching morphogenesis) pancreatic bud precursor cells
Defective SLC22A12 causes renal hypouricemia 1 (RHUC1)
Regulation of beta- cell development
Fibronectin matrix formation
Phase 1 - inactivation of fast Na channels
Organic anion transport
Hormone ligand- binding receptors
1.5 2.0 2.5 3.0
enrichment
pvalue
ER
TRP channels
MECP2 regulates neuronal receptors and channels
Collagen chain trimerization
Transcriptional Regulation by MECP2
Role of second messengers in netrin- 1 signaling
GABA receptor activation
2.0 2.2 2.4 2.6 2.8
enrichment
pvalue
2.8
2.6
2.4
2.2
gene_cou
5.0
7.5
10.0
12.5
15.0
Hmod
Erythrocytes take up oxygen and release carbon dioxide
Phosphorylation of the APC/C
'FAP2 (AP- 2) family regulates transcription of growth factors and their receptors
Polo- like kinase mediated events
O2/CO2 exchange in erythrocytes
Erythrocytes take up carbon dioxide and release oxygen
APC/C:Cdc20 mediated degradation of Cyclin B
Protein ubiquitination
Synthesis of active ubiquitin: roles of E1 and E2 enzymes
DNA methylation
2.2 2.4 2.6 2.8
enrichment
pvalue
TNBC
Non- integrin membrane- ECM interactions
Integrin cell surface interactions
Pre- NOTCH Transcription and Translation
Pre- NOTCH Expression and Processing
Laminin interactions
Extracellular matrix organization
Formation of Fibrin Clot (Clotting Cascade)
Defective F8 binding to von Willebrand factor
Defective F8 cleavage by thrombin
ECM proteoglycans
Signaling by NOTCH
2.0 2.5 3.0 3.5
enrichment
pvalue
3.5
3.0
2.5
gene_cou
1
2
3
4

Figure 2

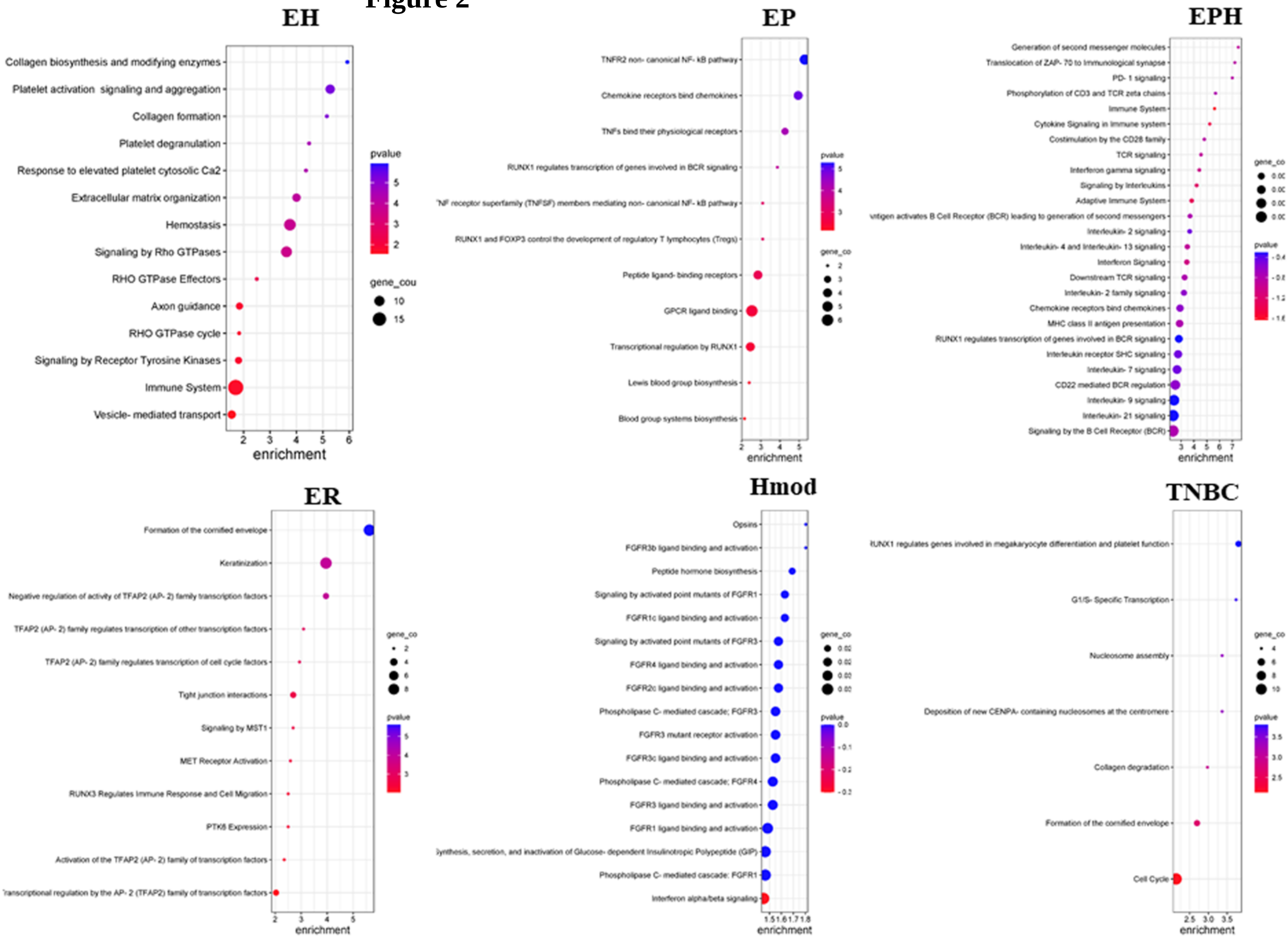
EH
Collagen biosynthesis and modifying enzymes
Platelet activation signaling and aggregation
Collagen formation
Platelet degranulation
Response to elevated platelet cytosolic Ca2
Extracellular matrix organization
Hemostasis
Signaling by Rho GTPases
RHO GTPase Effectors
Axon guidance
RHO GTPase cycle
Signaling by Receptor Tyrosine Kinases
Immune System
Vesicle- mediated transport
enrichment
pvalue
gene_cou
EP
TNFR2 non- canonical NF- kB pathway
Chemokine receptors bind chemokines
TNFs bind their physiological receptors
RUNX1 regulates transcription of genes involved in BCR signaling
'NF receptor superfamily (TNFSF) members mediating non- canonical NF- kB pathway
RUNX1 and FOXP3 control the development of regulatory T lymphocytes (Tregs)
Peptide ligand- binding receptors
GPCR ligand binding
Transcriptional regulation by RUNX1
Lewis blood group biosynthesis
Blood group systems biosynthesis
enrichment
pvalue
gene_co
EPH
Generation of second messenger molecules
Translocation of ZAP- 70 to Immunological synapse
PD- 1 signaling
Phosphorylation of CD3 and TCR zeta chains
Immune System
Cytokine Signaling in Immune system
Costimulation by the CD28 family
TCR signaling
Interferon gamma signaling
Signaling by Interleukins
Adaptive Immune System
ntigen activates B Cell Receptor (BCR) leading to generation of second messengers
Interleukin- 2 signaling
Interleukin- 4 and Interleukin- 13 signaling
Interferon Signaling
Downstream TCR signaling
Interleukin- 2 family signaling
Chemokine receptors bind chemokines
MHC class II antigen presentation
RUNX1 regulates transcription of genes involved in BCR signaling
Interleukin receptor SHC signaling
Interleukin- 7 signaling
CD22 mediated BCR regulation
Interleukin- 9 signaling
Interleukin- 21 signaling
Signaling by the B Cell Receptor (BCR)
enrichment
gene_co
pvalue
ER
Formation of the cornified envelope
Keratinization
Negative regulation of activity of TFAP2 (AP- 2) family transcription factors
TFAP2 (AP- 2) family regulates transcription of other transcription factors
TFAP2 (AP- 2) family regulates transcription of cell cycle factors
Tight junction interactions
Signaling by MST1
MET Receptor Activation
RUNX3 Regulates Immune Response and Cell Migration
PTK6 Expression
Activation of the TFAP2 (AP- 2) family of transcription factors
ranscriptional regulation by the AP- 2 (TFAP2) family of transcription factors
enrichment
gene_co
pvalue
Hmod
Opsins
FGFR3b ligand binding and activation
Peptide hormone biosynthesis
Signaling by activated point mutants of FGFR1
FGFR1c ligand binding and activation
Signaling by activated point mutants of FGFR3
FGFR4 ligand binding and activation
FGFR2c ligand binding and activation
Phospholipase C- mediated cascade; FGFR3
FGFR3 mutant receptor activation
FGFR3c ligand binding and activation
Phospholipase C- mediated cascade; FGFR4
FGFR3 ligand binding and activation
FGFR1 ligand binding and activation
Synthesis, secretion, and inactivation of Glucose- dependent Insulinotropic Polypeptide (GIP)
Phospholipase C- mediated cascade: FGFR1
Interferon alpha/beta signaling
enrichment
gene_co
pvalue
TNBC
RUNX1 regulates genes involved in megakaryocyte differentiation and platelet function
G1/S- Specific Transcription
Nucleosome assembly
Deposition of new CENPA- containing nucleosomes at the centromere
Collagen degradation
Formation of the cornified envelope
Cell Cycle
enrichment
gene_co
pvalue

Figure 3

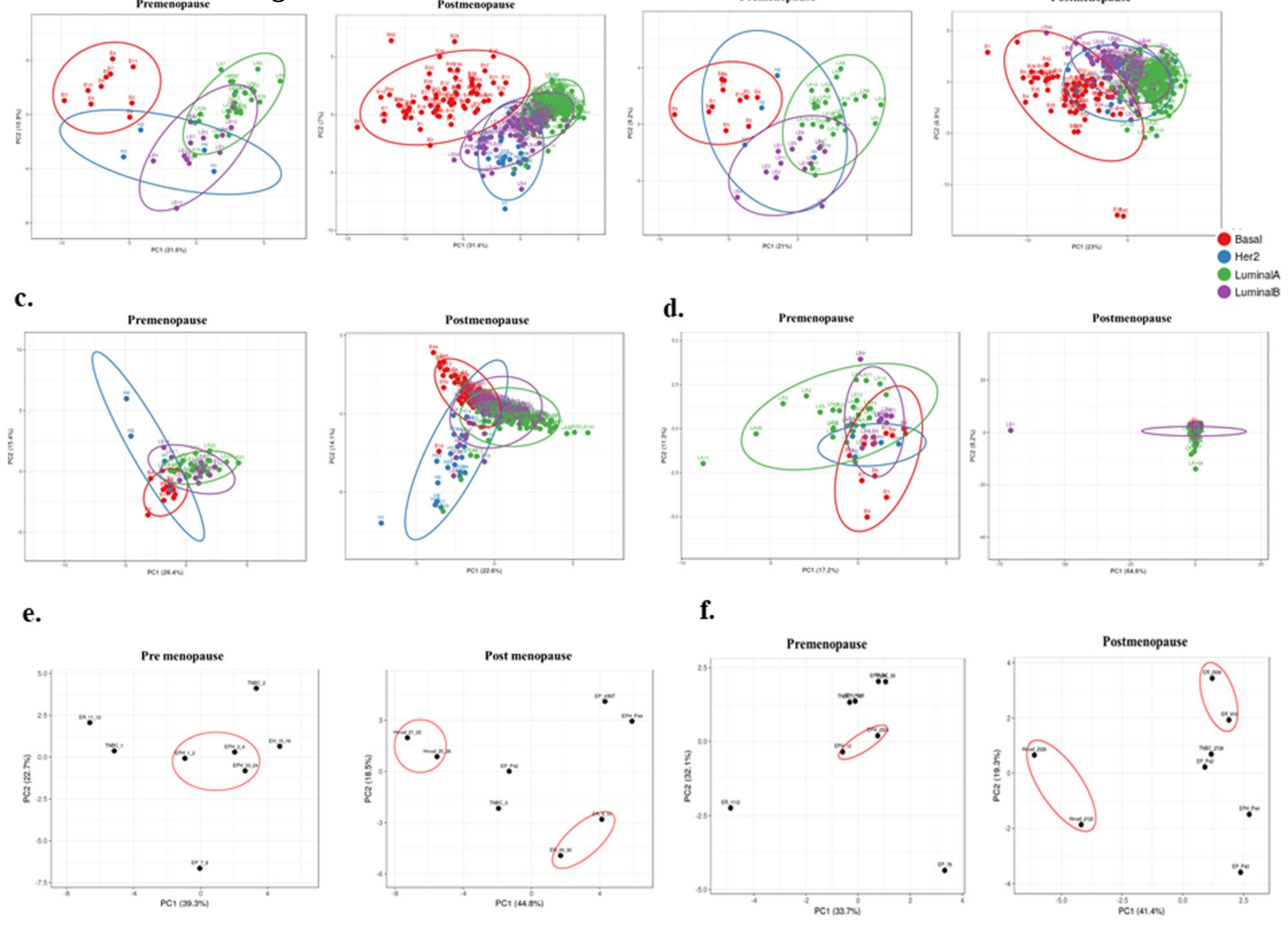
a.
Premenopause
Postmenopause
b.
Premenopause
Postmenopause
Basal
Her2
LuminalA
LuminalB
c.
Premenopause
Postmenopause
d.
Premenopause
Postmenopause
e.
Pre menopause
PC1 (39.3%)
PC2 (22.7%)
Post menopause
PC1 (44.8%)
PC2 (18.5%)
f.
Premenopause
PC1 (33.7%)
PC2 (32.1%)
Postmenopause
PC1 (41.4%)
PC2 (19.3%)

Figure 4

a.i.

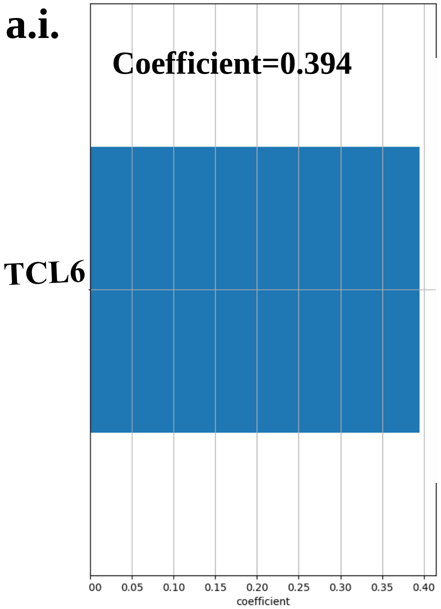

a.ii.

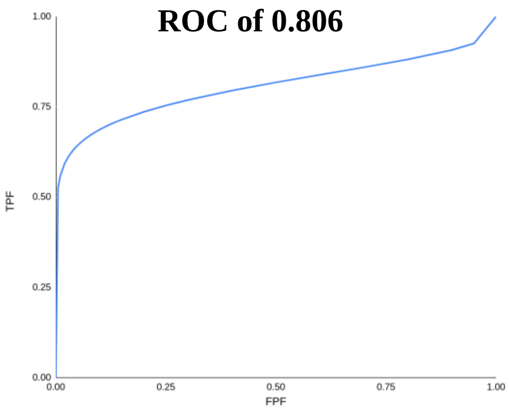

b.i.

best alpha 0.01397 and concordance index 0.675

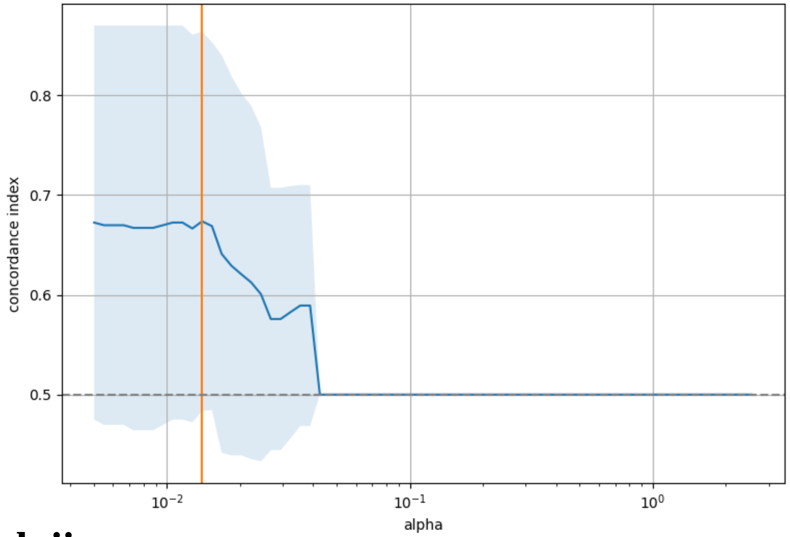

b.ii.

genes	coefficient
GRIN2A	-0.25049829
LRRC3B	-0.22998512
SNHG12	-0.23156815

b.iii.

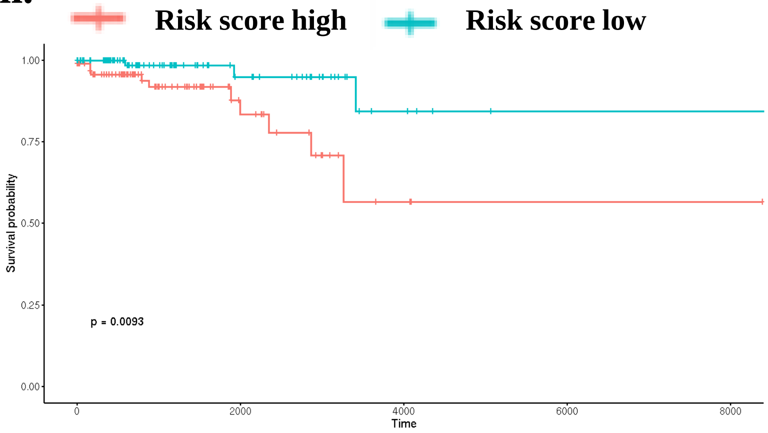

c.

Immune cells	SHEL	SLEH	Normal	SLEH_minus_normal	pval
B.cells.naive	0.108851	0.128379	0.216251	-0.087872581	0.2572
Dendritic.cells.activated	0	0	0.140128	-0.140128037	0.01451
Dendritic.cells.resting	0	0	0.082936	-0.082935945	0.01083
Eosinophils	0	0	0.080034	-0.080033756	0.01356
Macrophages.M0	0.104314	0.092069	0	0.092069157	0.08297
Macrophages.M1	0.074979	0.069413	0.219055	-0.149641834	0.02472
Macrophages.M2	0.346875	0.300617	0	0.300617106	0.003485
Mast.cells.activated	0	0	0.737936	-0.737935909	0.000144
Mast.cells.resting	0.079275	0.118992	0	0.118992087	4.96E-07
Monocytes	0.011752	0.017838	1.274367	-1.256529442	7.43E-07
NK.cells.resting	0.025894	0.019579	0.034498	-0.01491858	0.08713
Plasma.cells	0.024846	0.065263	0	0.065262809	2.21E-12
T.cells.CD4.memory.resting	0.169205	0.182743	1.534963	-1.352220067	0.000711
T.cells.CD4.naive	0	0	0.184019	-0.184019195	0.01589
T.cells.CD8	0.067805	0.057111	0	0.05711101	0.2735
T.cells.follicular.helper	0.070299	0.06477	0.120646	-0.055875352	0.1802
T.cells.gamma.delta	0	0	0.238416	-0.238415779	0.01345
T.cells.regulatory(Tregs)	0.024123	0.028881	0	0.028881356	6.66E-09

SHEL- SNHG12 high EPB41 low, SLEH - SNHG12 low EPB41 high
