# Supplementary material for: Transcriptomic profiling of Indian breast cancer patients revealed subtype-specific mRNA and lncRNA signatures
Source: Front Genet. 2022 Oct 25;13:932060. doi: 10.3389/fgene.2022.932060 (PMC9641000; doi:10.3389/fgene.2022.932060)
Supplement: Supplementary file 5 [file Table5.DOCX]

| **Patient No** | **Sample** | **Label** | **Subtype label** | **File size** | **Total Reads** | **Mapped reads** | **Coverage** |
| --- | --- | --- | --- | --- | --- | --- | --- |
| Patient 1 | Normal | P1 | EPH | 2.1 | 27436900 | 16368544 | 45.72816667 |
|  | Tumour | P2 |  | 5.4 | 98457512 | 83682154 | 164.0958533 |
| Patient 2 | Normal | P3 | EPH | 8.4 | 113058008 | 95318719 | 188.4300133 |
|  | Tumour | P4 |  | 3 | 41443626 | 34382861 | 69.07271 |
| Patient 3 | Normal | P5 | TNBC | 23.2 | 303036382 | 252092478 | 505.0606367 |
|  | Tumour | P6 |  | 3.8 | 49464644 | 44482267 | 82.44107333 |
| Patient 4 | Normal | P7 | EP | 13.4 | 177267404 | 116545958 | 295.4456733 |
|  | Tumour | P8 |  | 4 | 53492744 | 45825367 | 89.15457333 |
| Patient 5 | Normal | P9 | ER | 8.7 | 117412262 | 96537340 | 195.6871033 |
|  | Tumour | P10 |  | 4.6 | 62919394 | 57129891 | 104.8656567 |
| Patient 6 | Normal | P11 | ER | 9.6 | 142853800 | 115380208 | 238.0896667 |
|  | Tumour | P12 |  | 5.8 | 76358772 | 70085230 | 127.26462 |
| Patient 7 | Normal | P13 | EH | 0.066 | 8466 | 2561 | 0.01411 |
|  | Tumour | P14 |  | 5.1 | 68497432 | 60100670 | 114.1623867 |
| Patient 8 | Normal | P15 | EH | 4.1 | 55460172 | 45596481 | 92.43362 |
|  | Tumour | P16 |  | 5.7 | 76834102 | 35122831 | 128.0568367 |
| Patient 9 | Normal | P17 | TNBC | 3 | 39391050 | 33420229 | 65.65175 |
|  | Tumour | P18 |  | 0.2 | 2573894 | 2314149 | 4.289823333 |
| Patient 10 | Normal | P19 | Hmod | 1.6 | 20074426 | 16356287 | 33.45737667 |
|  | Tumour | P20 |  | 4.2 | 58847290 | 36207256 | 98.07881667 |
| Patient 11 | Normal | P21 | EPH | 4 | 54953952 | 33747534 | 91.58992 |
|  | Tumour | P22 |  | 1.9 | 25316472 | 23242694 | 42.19412 |
| Patient 12 | Normal | P25 | Hmod | 1.1 | 14950564 | 7585484 | 24.91760667 |
|  | Tumour | P26 |  | 3.4 | 47365906 | 23991453 | 78.94317667 |
| Patient 13 | Normal | P27 | TNBC | 5.9 | 81865694 | 73645673 | 136.4428233 |
|  | Tumour | P28 |  | 5.4 | 74632110 | 65204593 | 124.38685 |
| Patient 14 | Normal | P29 | ER | 3.8 | 52675726 | 23198523 | 87.79287667 |
|  | Tumour | P30 |  | 5.7 | 78357172 | 66484025 | 130.5952867 |
| Patient 15 | Tumour | P42 | EP | 19 | 215220038 | 155895747 | 358.7000633 |
| Patient 16 | Normal | P43N | EP | 6 | 78009304 | 53022219 | 130.0155067 |
|  | Tumour | P43T |  | 2.1 | 25033980 | 54022249 | 41.7233 |
| Patient 17 | Normal | P44N | EPH | 4.8 | 54121062 | 39615593 | 90.20177 |
|  | Tumour | P44T |  | 6 | 79673930 | 94199609 | 132.7898833 |

**Table 1:** Table showing sequencing details of all the samples. Total reads and file size for each sample is given.

**Supplementary Figure Legends**

**Figure 1:** Bubble plots showing significantly downregulated pathways in the six subtypes obtained from the Reactome database. Y-axis shows pathway terms and the x-axis is the pathway enrichment score. The size of the bubble represents the gene count of the pathway and the colour gradient of the bubble is based on the p-value

**Figure 2:** Bubble plots showing significantly upregulated pathways in the six subtypes obtained from the Reactome database. Y-axis shows pathway terms and the x-axis is the pathway enrichment score. The size of the bubble represents the gene count of the pathway and the colour gradient of the bubble is based on the p-value.

**Figure 3:** Principal Component Analysis of **a.** Pre- and post-menopause TCGA breast cancer patient samples with PAM50 **b.** Pre and post-menopause Indian breast cancer patient samples with Mamprint geneset. **c.** Pre and post-menopause TCGA breast cancer patient samples with Oncodx geneset. **d.** PCA plots showing segregation with selected mRNAs between pre- and post-menopause TCGA Breast Cancer patients **e.** PCA plots showing segregation with selected LncRNAs between pre and post-menopause Indian Breast cancer patients **f.** PCA plots showing segregation with selected mRNA-LncRNAs between pre and post-menopause Indian Breast cancer patients

**Figure 4**: **a.i.** Predicted features TCL6 with coefficient value of 0.394 at a best alpha 0.13153 from the lasso-cox regression for the HER2 TCGA dataset. **a,ii** ROC curve of the model with fitted ROC of 0.806 indicating a strong model. **b.i.** Alpha value selection plot for the in the Luminal A data set. Best alpha was found to be 0.00959 with a concordance index of 0.56. Concordance index being a measure of how strong the model is ranging from 0 to 1. **b.ii** Predicted features with coefficient values at a best alpha 0.00959 from the lasso-cox regression for the Luminal A TCGA dataset **b.iii.** Kaplan-Meier survival plot for high and low risk score obtained from risk score of 3 gene signature. **c.** Immune cell profiling using CIBERSORT for SNHG12 low and EPB41 high and normal samples
