# Supplementary material for: Transcriptomic profiling of Indian breast cancer patients revealed subtype-specific mRNA and lncRNA signatures
Source: Front Genet. 2022 Oct 25;13:932060. doi: 10.3389/fgene.2022.932060 (PMC9641000; doi:10.3389/fgene.2022.932060)
Supplement: Supplementary file 7 [file Table4.DOCX]

**Table 1:** Table depicting sample details of Indian Breast Cancer patients. Odd numbers are matched normals and even numbers are tumour samples. There are a total six subtypes (ER, EH, EP, EPH, Hmod and TNBC) classified based on the expression of estrogen receptor (ER), progesterone receptor (PR) and epidermal growth factor receptor (Her2). IDC (Invasive Ductal Carcinoma)

| **Gene** | **Primer sequence** | |
| --- | --- | --- |
|  | **Forward** | **Reverse** |
| **GAPDH** | 5’ **-** CCCTTCATTGACCTCAACTACAT-3’ | 5’-CTGGAGATGGTGATGGGATTT- 3’ |
| **BCL2** | 5’-AGAGACTCACCAGGGTCTGC-3’ | 5’- GCACTACCTGCGTTCTCCTC-3’ |
| **BRCA1** | 5’ CTGCCGTCCAAATTCAAGAAGT 3’ | 5’ CTTGTGCTTCCCTGTAGGCT 3’ |
| **TP53** | 5’-CTGCTTGCCACAGGTCTC-3’ | 5’-TGGATGGGTAGTAGTATGGAAG-3’ |
| **ALDH1A** | 5’-ACTTACCTGTCCTACTCA-3’ | 5’-CTTATCTCCTTCTTCTACCT-3’ |
| **CD44l** | 5’-CAGGTGGAAGAAGAGACCCAAA-3’ | 5’-GGATGAAGGTCCTGCTTTCCTT |
| **CD44s** | 5’-TCCAACACCTCCCAGTATGACA-3’ | 5’-GGCAGGTCTGTGACTGATGTACA-3’ |
| **HOTAIR** | 5’- GGTAGAAAAAGCAACCACGAAGC-3’ | 5’-ACATAACCTCTGTCTGTGAGTGCC-3’ |

**Table 2:** Sequences of primers used for RTPCR validation
